# Synthesis of phosphoramidites of isoGNA, an isomer of glycerol nucleic acid

**DOI:** 10.3762/bjoc.10.220

**Published:** 2014-09-08

**Authors:** Keunsoo Kim, Venkateshwarlu Punna, Phaneendrasai Karri, Ramanarayanan Krishnamurthy

**Affiliations:** 1 Department of Chemistry, The Scripps Research Institute, 10550 North Torrey Pines Rd, La Jolla, CA 92037, USA

**Keywords:** acyclic nucleic acids, glycerol nucleic acids, isoGNA, oligonucleotides, phosphoramidites

## Abstract

IsoGNA, an isomer of glycerol nucleic acid GNA, is a flexible (acyclic) nucleic acid with bases directly attached to its linear backbone. IsoGNA exhibits (limited) base-pairing properties which are unique compared to other known flexible nucleic acids. Herein, we report on the details of the preparation of isoGNA phosphoramidites and an alternative route for the synthesis of the adenine derivative. The synthetic improvements described here enable an easy access to isoGNA and allows for the further exploration of this structural unit in oligonucleotide chemistry thereby spurring investigations of its usefulness and applicability.

## Introduction

**Figure 1:**
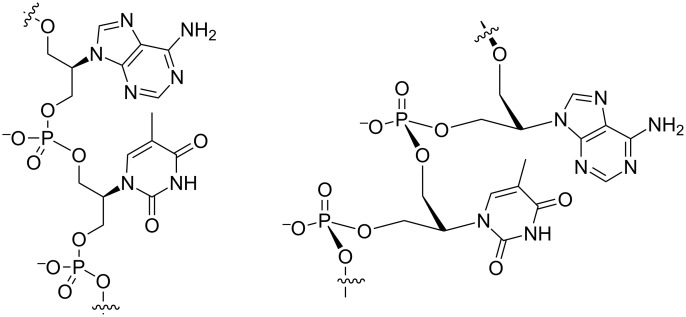
A constitutional and conformational (idealized) representation of isoGNA, an isomer of glycerol nucleic acid.

Acyclic nucleic acids have garnered a lot of attention and are becoming an important component in nucleic acid chemistry [1–3]. Lately, there has been an explosion in the number of investigated candidates [4–7]. We have recently reported on the base-pairing properties of isoGNA, an isomer of glycerol derived nucleic acid (Figure 1) [8]. Our motivation was driven by the question “how structurally simple and minimal can an oligonucleotide be and still exhibit base-pairing?” When we studied isoGNA we were surprised to find that the base-pairing properties were unpredictably different from other closely related acyclic nucleic acids [8]. This indicated that the nature of the backbone exerts a strong influence on the disposition of the nucleobases, and there seems to be no straightforward correlation between the nature of the backbone and base-pairing properties [9].

We are continuing our investigation of isoGNA oligonucleotides within the context of chimeric sequences and collaborative intercalation studies and thus have a continuing need for the synthesis of isoGNA building blocks. While iso-glycerol nucleosides are known [10–15], the published synthetic routes and efficiencies of these vary widely and primarily depend on the nature of the nucleobase. Herein, we detail our synthetic efforts and the current improvements for the synthesis of all four isoGNA nucleosides and their phosphoramidites.

## Results and Discussion

### Initial synthetic routes

**Scheme 1:**
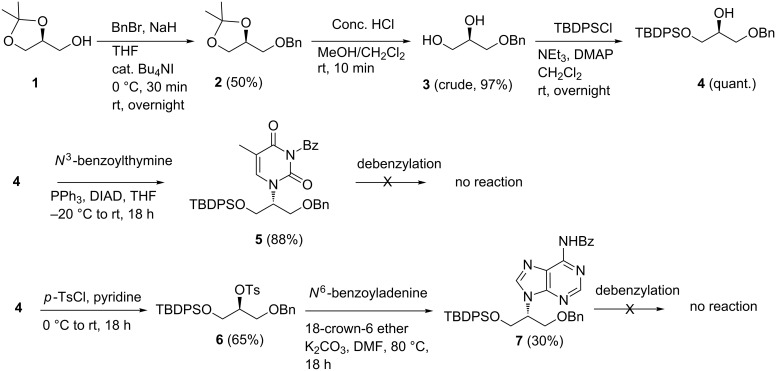
Initial routes towards the synthesis of iso-glycerol nucleosides.

We started with commercially available (*S*)-solketal (**1**), which was protected as the benzyl ether **2**. Ketal deprotection followed by the silylation of the primary hydroxy group with *tert*-butyldiphenylsilyl chloride provided the substrate **4**, which is suitable for the introduction of the canonical nucleobases by a S_N_2 displacement reaction. At this point, we chose the Mitsunobu reaction (Scheme 1), which has been widely employed [16]. The reaction with the *N*^3^-benzoyl protected thymine delivered **5** in good yield. However, the reaction with *N*^6^-benzoyladenine proved problematic and no product was detected. Therefore, in the adenine case, we tried a direct S_N_2 reaction of *N*^6^-benzoyladenine with tosyl derivative **6**, which was only moderately successful (30% yield of **7**). Unexpectedly, in both the thymine and adenine cases, the subsequent debenzylation reaction failed under a variety of conditions (H_2_, 10% Pd/C, MeOH or EtOAc; Pd/C, DMF, cyclohexadiene; 10% Pd/C, NH_4_HCOO, acetone, reflux; 10% Pd-black, DMF, cyclohexadiene; (CH_3_)_3_SiI, CHCl_3_, 25 °C), for reasons that were not obvious to us. Consequently, we abandoned this approach and pursued a different protecting group approach.

**Scheme 2:**
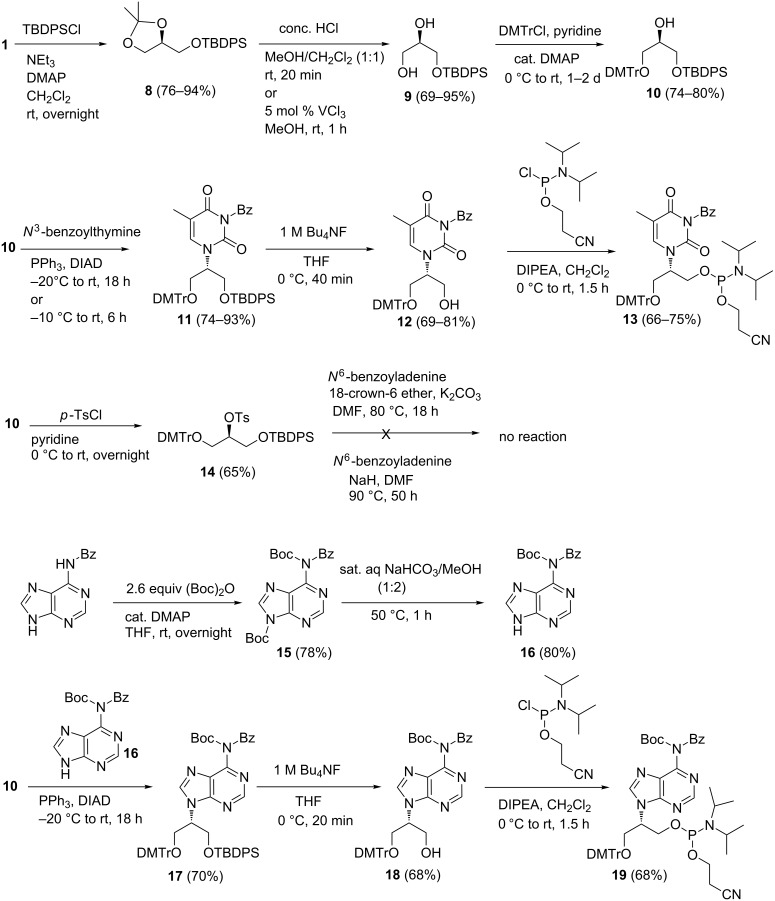
Preparation of thymine and adenine containing phosphoramidites of isoGNA.

We silylated **1** to afford **8** whose ketal was deprotected followed by tritylation to give the derivative **10**, which is expected to be suited for the introduction of nucleobases by an S_N_2 reaction (Scheme 2). Once again, the Mitsunobu reaction with thymine proceeded smoothly. However, the reaction with *N*^6^-benzoyladenine proved problematic and did not proceed even with the tosyl derivative **14**. The solubility issues with benzoylated adenine and the bulk of the O-TBDPS group may be the underlying reasons behind this difficulty. Therefore, we proposed to use a doubly *N*^6^-protected adenine, the *N*^6^-benzoyl-Boc derivative **16**, which was synthesized starting from *N*^6^-benzoyladenine in two steps. *N*^6^-Benzoyladenine was treated with 2.6 equiv (Boc)_2_O to afford *N*^6^-benzoyl-*N*^6^,*N*^9^-di-Boc-adenine **15**; sat. aq NaHCO_3_/MeOH (1:2) conditions cleaved the *N*^9^-Boc group of **15** specifically [17–18] to yield **16**. The use of **16** in the subsequent Mitsunobu reaction afforded 68% of the desired product **17**. With derivatives **11** and **17** in hand we proceeded to prepare the corresponding phosphoramidite derivatives **13** and **19**, respectively, under standard conditions as outlined in Scheme 2.

**Scheme 3:**
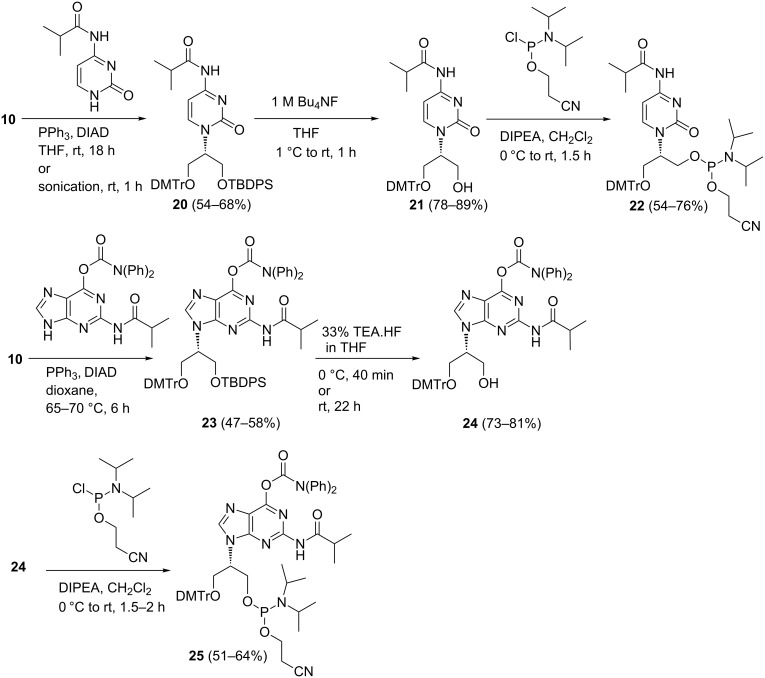
Preparation of guanine- and cytosine-containing phosphoramidites of isoGNA.

We proceeded to prepare the cytosine- and guanine-containing phosphoramidite by using the tritylated intermediate **10** as the starting point (Scheme 3). In the cytosine series we found that the normal *N*^4^-benzoyl or *N*^4^-acetyl protected cytosine did not give satisfactory yields under the Mitsunobu conditions. The best yields were achieved with *N*^4^-isobutyrylcytosine as the nucleophile [19–21]. In the guanine series, the *N*^2^-isobutyryl, *O*^6^-diphenylcarbamoyl protected guanine worked well (Scheme 3) [22].

With all the four phosphoramidites in hand we proceeded to the automated synthesis of the oligomers, which proceeded smoothly with ≥95% coupling efficiency as determined by trityl assays. It was at the stage of the removal of the protecting groups to afford the free oligos that we encountered a severe problem with the adenine derivative. While the aqueous ammonia/methylamine base deprotection removed all base labile protecting groups (acetate, isobutyrate, diphenylcarbamoyl groups), the *N*^6^-Boc protecting group on adenine was impervious. We tried different acidic conditions (trifluoroacetic acid, acetic acid with heat, AlCl_3_ [23–25]), but all of these conditions started to degrade the oligomer without completely removing the *tert*-Boc group (as seen by MALDI–TOF mass spectral data in combination with ion-exchange HPLC monitoring) [8].

### Improved synthesis of adenine-isoGNA building block

**Scheme 4:**
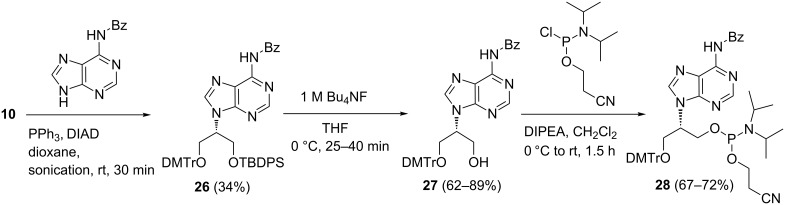
Preparation of adenine containing phosphoramidite of isoGNA.

We went back in our synthesis in order to address the adenine protecting group problem and to re-work the inefficient Mitsunobu reaction of *N*^6^-benzoyladenine. We explored various conditions (different solvents, temperature etc.) and found that the sonication [26] of the reaction mixture by using *N*^6^-benzoyladenine in anhydrous dioxane led to respectable yields (34–36%) of **26** accompanied by a (unidentified) byproduct (Scheme 4). Without sonication no product **26** was formed (by TLC).

However, the yield of the sonication reaction varied capriciously and the isolation of the compound was complicated by interference from the DEAD side-products during column chromatographic purification. We did manage to get enough amounts of **26** to prepare the phosphoramidite **28**, and proceeded with the oligomer synthesis for our studies. However, this was far from satisfactory, and the continued demand for isoGNA oligomers within our group and our collaborative work prompted us to look for an improvement of the preparation of the adenine building block. Considering the possible reasons for the inefficient reaction of the protected adenine derivatives in the Mitsunobu reaction, we focused on the modification of adenine in order to improve its solubility.

We considered three different derivatives of adenine based on increased solubility: 6-chloroadenine has been utilized to overcome the solubility and regioselectivity (*N*^7^ versus *N*^9^ nucleosidation) issues. Yet, the conversion of the chloro to the NH_2_ group is not efficient enough to consider this option an attractive one [27]. The second option was the *N*^6^,*N*^6^-dibenzoyladenine derivative, but this compound was found to be highly unstable for isolation and handling (one of the *N*^6^-benzoyl group falls off very easily). The third option was the use of the *N*^6^,*N*^6^-di-Boc-adenine derivative, even though this entails the difficulty in removing these Boc groups, which are incompatible with the oligonucleotide backbone. Therefore, we considered the option of removing these Boc groups after the Mitsunobu reaction is performed, and to introduce the *N*^6^-benzoyl group before the oligo stage.

The Schneller group has reported that *N*^6^-amino-di-Boc-protected adenine undergoes an efficient reaction under classic Mitsunobu conditions as a result of its increased solubility [28]. The regioselective attack at the *N*^9^-position is a consequence of the steric bulk of the di-Boc protection at the *N*^6^-position, which renders the *N*^7^-position inaccessible. As expected, a high yield was observed under mild conditions with substrates containing a secondary alcohol [28].

**Scheme 5:**
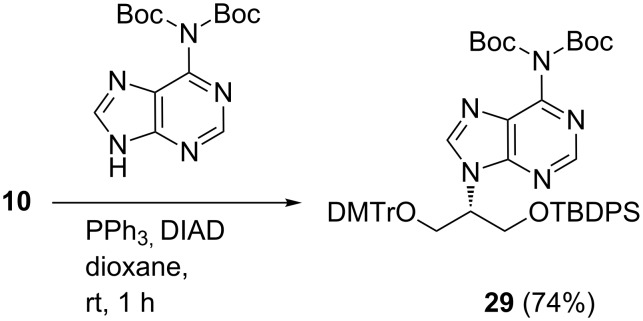
Mitsunobu reaction with the di-Boc-adenine.

Di-Boc protected adenine was readily prepared via protection and followed by selective deprotection according to a literature procedure [17–18]. The Mitsunobu reaction between the di-Boc-adenine and **10** was investigated, and it was found that dioxane as a solvent almost doubled the yield (74%) compared to THF (36%; Scheme 5). Interestingly, sonication afforded no tangible advantage. Doubling the amount of DIAD or di-Boc-adenine or PPh_3_ did not give increased yields. The desired product **29** was easily purified on silica gel due to its lower polarity (when compared with the *N*^6^-benzoyladenine reaction).

**Scheme 6:**
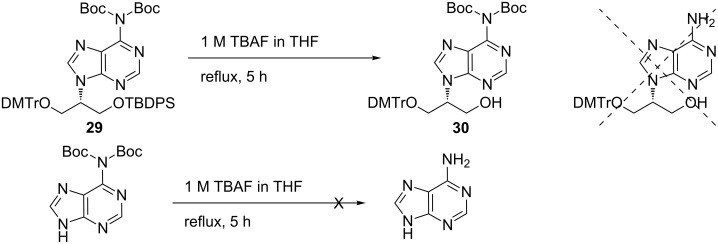
An attempt to remove both Boc and silyl groups simultaneously.

Initially, we tried to simultaneously deprotect both Boc groups and the TBDPS group in the presence of TBAF in a one-pot reaction [29]. However, only the TBDPS group was removed and a quantitative amount of mono-protected compound **30** was produced (Scheme 6). The fluoride ion was not effective enough to remove at least one of the Boc groups in **29**. We checked the deprotection conditions for the removal of the Boc moiety by fluoride ions on the model substrate *N*^6^,*N*^6^-di-Boc-adenine, and found that it was very challenging because free adenine was not afforded even under reflux with 1 M TBAF for 4 days.

Finally, we decided to remove the Boc groups on adenine with trifluoroacetic acid (TFA), even though we also expected the loss of the DMTr group on **29**. When **29** was treated with 20% of TFA in CH_2_Cl_2_, a mixture of **32** and **33** formed after 4 h. The TBDPS moiety was found to be stable under these conditions (Table 1, entry 1). 50% of TFA in CH_2_Cl_2_ completely removed both Boc groups on adenine at either 4 °C or rt to afford the desired **33** in 87% yield.

**Table 1:** Result of Boc deprotection of **29** with TFA.

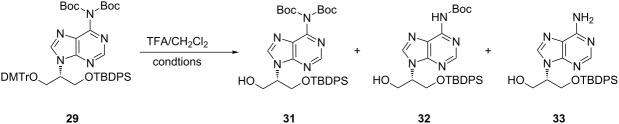						

Entry	Ratio of TFA (%)	Temp.^a^		Time (h)	Ratio (%)^b^ (**31**:**32**:**33**)	Yield

1	20	0 °C to rt		4	0:33:66	–
2	50	0 °C to 4 °C		2	0:20:80	–
				4	0:0:100	–
3^c^	50	0 °C to rt		2	0:0:100	87%

^a^TFA (99%) was added under ice-bath (−2 to 2 °C) cooling. ^b^The ratio is roughly calculated by the intensity of visualized spots on TLC by UV lamp. ^c^This reaction was performed on a large scale.

**Scheme 7:**
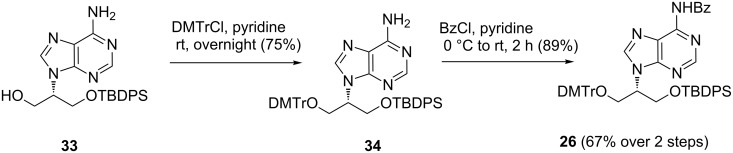
The synthesis of **26** via tritylation and benzoylation.

The trityl group was reintroduced on **33** under standard conditions to give **34**, and subsequent benzoylation afforded **26** in overall good yields (Scheme 7). The NMR spectra of **26** were in good agreement with those data obtained previously [8] via Mitsunobu reaction with *N*^6^-benzoyladenine.

Overall, **26** was afforded in 43% yield from the Mitsunobu reaction of **10** with *N*^6^,*N*^6^-di-Boc-adenine. In addition to the slight improvement compared to the direct Mitsunobu reaction of **10** with *N*^6^-benzoyladenine, this protocol allows for the synthesis of large amounts of **26** with an exact control of the regioselectivity at the *N*^9^-position of adenine.

Having solved the “adenine problem”, we focused on each of the remaining steps and optimized the synthesis of other isoGNA phosphoramidites. For example, the deprotection of **8** to **9** was initially achieved by the treatment with conc. HCl. However, in scaling up the reaction, the loss of the silyl group was found to occur. Therefore, we sought an alternative route and found that the deprotection mediated by vanadium trichloride gave the desired product **9** cleanly in large-scale reactions [30]. The desilylation of intermediates **11**, **20** and **26** was effected by the treatment of 1 M TBAF in THF at 0 °C to room temperature. In the case of guanine derivative **23**, we observed that the use of the triethylamine·HF complex instead of TBAF was preferable at room temperature. The free alcohols **12**, **18**, **21** and **24** were phosphitylated under standard conditions to obtain the phosphoramidites suitable for the automated solid phase synthesis.

## Conclusion

We have described herein improvements to and the optimization of the synthesis of isoGNA phosphoramidite building blocks. We observed that the Boc protecting group on adenine while not being compatible with oligonucleotide chemistry, can nevertheless be used as a temporary protecting group, which is extremely useful for increasing the solubility and regioselectivity of adenine in the Mitsunobu reaction. The optimized procedures described here allowed us to access a stable supply of isoGNA phosphoramidites for further investigation in our laboratory.

## Supporting Information

File 1 Experimental procedures.

File 2 ^1^H, ^13^C and ^31^P NMR spectra.
