# Supplementary material for: Synthesis of phosphoramidites of isoGNA, an isomer of glycerol nucleic acid
Source: Beilstein J Org Chem. 2014 Sep 8;10:2131–8. doi: 10.3762/bjoc.10.220 (PMC4168771; doi:10.3762/bjoc.10.220)
Supplement: File 1 — Experimental procedures. [file Beilstein_J_Org_Chem-10-2131-s001.pdf]

Supporting Information

for

Synthesis of phosphoramidites of isoGNA, an isomer of glycerol nucleic acid

Keunsoo Kim, Venkateshwarlu Punna, Phaneendrasai Karri and Ramanarayanan Krishnamurthy*

Address: Department of Chemistry, The Scripps Research Institute, 10550 North Torrey Pines Rd, La Jolla, CA 92037, USA

Email: Ramanarayanan Krishnamurthy - rkrishna@scripps.edu

* Corresponding author

Experimental procedures

Experimental

General experimental: All the solvents for reactions were anhydrous (*DriSolv*®) and were used as purchased from *EMD chemicals*. Thin layer chromatography (TLC) was performed on silica gel 60Å F_{254} (*Whatman*, type Al Silica G/UV, aluminium backing, 250 mm layer); visualization by UV lamp and/or charring solution of phosphomolybdic acid (PMA) in ethanol. Column chromatography was performed on silica gel 60 (40-63 mm, 230-400 mesh, *EM science*). All chromatography solvents were ACS grade and were used as purchased through *VWR* or *Fisher*.

NMR spectra were measured on Bruker 400-600 MHz using deuterated solvents from *Cambridge Isotope Labs*. Chemical shifts δ are quoted in ppm and are adjusted to the residual deuterated solvent peak as reference. Coupling constants are given in Hertz (Hz). Mass spectra were recorded using ESI-MS with *Micromat-LCT* with m/z (intensity in %).

(*R*)-*t*-Butyl((2,2-dimethyl-1,3-dioxolan-4-yl)methoxy)diphenylsilane (**8**) [1]**:** To a solution of (S)-solketal **1** (10.0 g, 75.8 mmol) and 4-dimethylaminopyridine (700 mg, 5.8 mmol) in anhydrous CH_2Cl_2 (500 mL) was added triethylamine (15.8 mL, 113.2 mmol) in an one portion of syringe under N_2 atmosphere. To the colorless clean solution was added *t*-butyldiphenyl chlorosilane (26.2 mL, 100.6 mmol) rapidly drop-wise (~ 5 minutes) with stirring at room temperature under N_2 atmosphere. The reaction mixture was vigorously stirred overnight at room

---

[1] Leftheris, K.; Goodman, M. *Synthesis*, **1989**, 564-565; Liang, C.; Lee, D. W.; Newton, M. G.; Chu, C. K. *J. Org. Chem.* **1995**, *60*, 1546-1553.

temperature. And then, the slightly pale yellow homogeneous mixture was washed with aqueous, saturated $NaHCO_3$ solution (300 mL) and H_2O (300 mL). The organic layer (CH_2Cl_2) was dried over Na_2SO_4 and the filtrate was concentrated in vacuo. The yellow sticky gel was purified by silica gel column chromatography (column dimensions ID, 6 cm x length, 35 cm; flow rate, controlled by pressure; loading silica gel, 300 g) using *n*-hexane: EtOAc (9:1) as eluent to afford 21.2 g (76%) of **8** as syrup. TLC (SiO_2), R_f = 0.70, *n*-hexane:EtOAc = 9:1. ^{1}H NMR (600 MHz, $CDCl_3$): δ 7.70 – 7.36 (m, 10H), 4.27 – 4.18 (m, 1H, CH), 4.11 (dd, *J* = 12.0, 1H, CH_2), 3.94 (dd, *J* = 12.0, 9.0 Hz, 1H, CH_2), 3.76 (dd, *J* = 15.6, 6.6 Hz, 1H), 3.68 (dd, *J* = 15.6, 9.6 Hz, 1H), 1.41 (s, 3H, CH_3), 1.38 (s, 3H, CH_3), 1.07 (s, 9H, CH_3). ^{13}C NMR (150 MHz, $CDCl_3$): δ 136.00, 135.23, 133.77, 130.15, 130.14, 128.12, 109.61, 67.25, 64.98, 27.22, 27.14, 26.98, 25.90,19.65.

(*R*)-3-(*t*-Butyldiphenylsilyloxy)propane-1,2-diol (**9**) [2]**:** To a solution of the silyl compound **8** (18.2 g, 49.3 mmol) in anhydrous CH_3OH (300 mL) was added 5 mol% of vanadium(III) chloride (400 mg, 2.5 mmol) in an one portion. The homogeneous pale green solution was vigorously stirred for 1 hour at room temperature under N_2 atmosphere and the color of the reaction mixture turned dark green solution. After checking TLC showing that the starting material was completely consumed, the reaction mixture was concentrated in vacuo and the yellow sticky gel was dissolved in CH_2Cl_2 (200 mL). The organic layer was

---

[2] Dey, S.; Garner, P. *J. Org. Chem*. **2000**, *65*, 7697-7699.

washed with H_2O (100 mL) and brine (100 mL) and dried over Na_2SO_4. The filtrate was concentrated in vacuo and the yellow sticky gel was purified by silica-gel column chromatography (column dimensions ID, 6 cm x length, 35 cm; flow rate, controlled by pressure; loading silica gel, 250 g) using *n*-hexane:EtOAc (2:1 to 3:2 to 2:3) as eluent to afford 11.0 g (69%) of **9** as white foam. TLC (SiO_2), R_f = 0.55, *n*-hexane:EtOAc = 2:1.^{1}H NMR (600 MHz, DMSO-d_6): δ 7.67 – 7.42 (m, 10H, ArH), 4.66 (d, *J* = 4.8 Hz, 1H, OH), 4.48 (t, *J* = 5.6 Hz, 1H, CH), 3.66 – 3.53 (m, 3H), 3.49 (dt, *J* = 10.7, 5.3 Hz, 1H), 3.39 (dt, *J* = 10.9, 5.4 Hz, 1H), 1.00 (s, 9H, CH_3). ^{13}C NMR (150 MHz, DMSO-d_6): δ 135.09, 133.27, 129.74, 127.81, 72.05, 65.40, 62.80, 26.66, 18.86

(*R*)-1-(Bis(4-methoxyphenyl)(phenyl)methoxy)-3-((*tert*-butyldiphenylsilyl)oxy)propan-2-ol (**10**)**:** To a solution of the diol compound **9** (11.0 g, 33.3 mmol) in anhydrous pyridine (300 mL) was added 4-dimethylaminipyridine (290 mg, 2.3 mmol) and 4,4-dimethoxytrityl chloride (13.7 g, 40.7 mmol) at 0 °C under N_2 atmosphere. The ice-bath was removed and the reaction mixture was allowed to warm to room temperature and stirred for 1 day under N_2 atmosphere. The yellowish reaction mixture was turned into more dark yellow solution. After checking TLC showing that the starting material was completely consumed, the reaction mixture was concentrated in vacuo. The residue was taken up in CH_2Cl_2 (200 mL), and then washed with aqueous, saturated $NaHCO_3$ (100 mL), brine (100 mL) and the organic layer was dried over Na_2SO_4. The filtrate was concentrated in vacuo and the dark yellowish gum

was purified by silica-gel column chromatography (column dimensions ID, 4.5 cm x length, 45 cm; flow rate, controlled by gravity; loading silica gel, 250 g) using *n*-hexane:EtOAc (4:1) as eluent to afford 15.6 g (74 %) of **10** as white foam. TLC (SiO_2), R_f = 0.7, *n*-hexane:EtOAc = 4:1. ^{1}H NMR (600 MHz, $CDCl_3$): δ 8.65 (s, 1H, NH), 7.65 – 6.81 (m, 23H, ArH), 3.94 – 3.88 (m, 1H, CH), 3.80 (d, J = 2.0 Hz, 6H, OCH_3), 3.83 – 3.74 (m, 2H, CH_2), 3.28 (dd, J = 9.6, 6.0 Hz, 1H, H of CH_2), 3.22 (dd, J = 9.6, 5.4 Hz, 1H, H of CH_2), 1.01 (s, 9H, ^{t}Bu). ^{13}C NMR (150 MHz, $CDCl_3$): δ 158.86, 136.48, 133.62, 130.46, 130.14, 129.54, 128.60, 128.19, 128.14, 127.89, 127.47, 127.12, 113.30 (all ArH), 86.47 (C-DMTr), signal for (CH) not seen, weak signals for 66.51 (CH_2) and 64.99 (CH_2), 52.64 (OCH_3), 27.23 (C-^{t}Bu), 19.57 (CH_3-^{t}Bu).

(*S*)-3-Benzoyl-1-(1-(bis(4-methoxyphenyl)(phenyl)methoxy)-3-((*tert*-butyldiphenylsilyl)oxy)propan-2-yl)-5-methylpyrimidine-2,4(1*H*,3*H*)-dione (**11**): To a heterogeneous solution of the alcohol compound **10** (4.06 g, 6.4 mmol), N^3-benzoyl-thymine (1.63 g, 7.0 mmol), and triphenylphosphine (PPh_3) (1.84 g, 7.0 mmol) in anhydrous 1,4-dioxane (30 mL) was added drop wise (~ 10 minutes) diisopropyl azodicarboxylate (DIAD) (1.6 mL, 7.7 mmol) at -10 ^{o}C under N_2 atmosphere. During the dropping half of the amount of DIAD, the reaction mixture was turned into a clean yellow solution. Once DIAD was completely added, the reaction mixture was heterogeneous. The reaction mixture was allowed to warm to room temperature and turned into clean yellow solution again. The reaction mixture was stirred for 6 hours, at which point no further

improvement was observed in the product formation by TLC analysis. The reaction mixture was concentrated in vacuo. The sticky yellow gel residue was purified by silica-gel column chromatography (column dimensions ID, 4 cm x length, 45 cm; flow rate, controlled by gravity; loading silica gel, 150 g) using *n*-hexane:EtOAc (7:3) as eluent to afford 4.01 g (74%) of **11** as white foam. TLC (SiO_2), R_f = 0.56, *n*-hexane:EtOAc = 7:3. ^{1}H NMR (600 MHz, $CDCl_3$): δ 7.91 – 6.82 (m, 28H, ArH, C(6)H), 4.96 – 4.88 (br s, 1H, CH), 3.91 (d, *J* = 6 Hz, 2H, CH_2), 3.73 (s, 6H, OCH_3), 3.55 – 3.45 (m, 2H, CH_2), 1.84 (s, 3H, C(5)CH_3), 1.00 (s, 9H, ^{t}Bu). ^{13}C NMR (150 MHz, DMSO-d_6): δ 170.33 (CO), 163.19 (CO), 158.65, 150.84, 145.45, 136.25, 135.99, 135.90, 135.85, 133.24, 133.17, 132.07, 130.91, 130.88, 130.80, 130.49, 130.30, 128.78, 128.74, 128.29, 127.63, 114.07, 109.20, 86.49 (C-DMTr), 62.30 (CH_2), 61.43 (CH_2), 60.62 (CH), 55.89 (OCH_3), 27.30 (C), 19.55 (C(5)CH_3), 12.74 (CH_3). ESI-MS (+ve): 205.11 (90%), 409.22 (100%), 431.20 (75%), 845.34 (M+H, 2%), 867.33 (M+Na, 10%).

(*R*)-3-Benzoyl-1-(1-(bis(4-methoxyphenyl)(phenyl)methoxy)-3-hydroxypropan-2-yl)-5-methylpyrimidine-2,4(1*H*,3*H*)-dione (12): To a solution of the N^3-benzoyl-thymine derivative **11** (314 mg, 0.4 mmol) in anhydrous THF (30 mL) was added slowly drop wise (~ 2 minutes) 1M tetrabutylammonium fluoride in THF (1.2 mL) under ice-bath (0 °C) and N_2 atmosphere. After removing the ice-bath, the colorless clean reaction mixture was allowed to warm to room temperature and vigorously stirred for 40 minutes, at which point starting material was completely consumed and new spots were observed by TLC

monitoring. CH_2Cl_2 (20 mL) was added into the reaction mixture. The organic layer was washed with H_2O (20 mL) and brine (20 mL), and then dried over Na_2SO_4. The filtrate was concentrated in vacuo. The yellow foam residue was purified by silica gel column chromatography (column dimensions ID, 2 cm x length, 30 cm; flow rate, controlled by pressure; loading silica gel, 30 g) using *n*-hexane:EtOAc (4:1 including 1% TEA) as eluent to afford 156 mg of **12** (69%) as white foam. TLC (SiO_2), R_f = 0.68, *n*-hexane:EtOAc = 1:4. ^{1}H NMR (600 MHz, $CDCl_3$): δ 7.92 – 6.86 (d, *J* = 8.5 Hz, 20H, ArH, C(6)H), 4.76 – 4.67 (br s, 1H, CH), 4.05 – 3.86 (m, 2H, CH_2), 3.82 (s, 6H, OCH_3), 3.61 – 3.46 (m, 2H, CH_2), 1.94 (s, 3H, CH_3). ^{13}C NMR (150 MHz, $CDCl_3$): δ 169.68 (CO), 163.46 (CO), 159.39, 151.17, 144.89, 139.92 135.83, 135.54, 132.32, 131.09, 130.60, 129.81, 129.29, 128.73, 128.58, 128.01, 127.78, 114.03, 110.64, 87.45 (C-DMTr), 62.41 (CH_2), 62.02 (CH_2), 58.81 (CH), 55.93 (OCH_3), 13.17 (CH_3). ESI-HRMS (+ve): 186.22 (100%), 303.14 (50%), 409.22 (40%), expected 607.2439, found 607.2468 (M+H, 2%), expected 629.2258, found 629.2289 (M+Na, 40%).

(*S*)-2-(3-Benzoyl-5-methyl-2,4-dioxo-3,4-dihydropyrimidin-1(2*H*)-yl)-3-(bis(4-methoxyphenyl)(phenyl)methoxy)propyl (2-cyanoethyl) diisopropylphosphoramidite (**13**): A solution of the 3’-OH-thymine **12** (607 mg, 1.0 mmol) and diisopropylethylamine (1.0 mL, 5.7 mol) in anhydrous CH_2Cl_2 (30 mL) was added 2-cyanoethyl *N*,*N*-diisopropylchlorophosphoramidite (300 µL, 1.27 mmol) at 0 ^{o}C under N_2 atmosphere. The yellowish reaction mixture was allowed to warm to room temperature and stirred for 1.5 hour, at which point

starting material was remained based on TLC monitoring. An additional 2-cyanoethyl *N*,*N*-diisopropylchlorophosphoramidite (100 μL, 0.42 mmol) was added to complete the reaction. After 30 minutes, no further improvement was observed in the product formation by TLC analysis. The reaction mixture was washed with aqueous, saturated $NaHCO_3$ (30 mL) and the organic layer (CH_2Cl_2) was dried over Na_2SO_4. The filtrate was concentrated in vacuo. The yellow foam was purified by silica gel column chromatography (column dimensions ID, 3 cm x length, 30 cm; flow rate, controlled by pressure; loading silica gel, 50 g) using n-hexane:EtOAc (3:2 including 2% TEA) as eluent to afford 605 mg of **13** (75%) as white foam. TLC (SiO_2), R_f = 0.75, *n*-hexane:EtOAc = 1:1. ^{1}H NMR (600 MHz, $CDCl_3$): δ 7.93 – 6.85 (m, 18H, ArH, C(6)H), 5.03-4.91 (br s, 1H, CH), 4.03 – 3.84 (m, 2H, CH_2), 3.82 (s, 6H, OCH_3), 3.84 – 3.68 (m, 2H, CH_2), 3.61 – 3.47 (m, 4H, CH_2, $CH(CH_3)_2$), 2.62 – 2.52 (m, 2H, CH_2CN), 1.93 (s, 3H, C(5)CH_3 of one diastereomer), 1.92 (s, 3H, C(5)CH_3 of one diastereomer), 1.19 (d, J = 6.9 Hz, 6H, $C(CH_3)_2$), 1.12 (d, J = 6.9 Hz, 6H, $C(CH_3)_2$). ^{13}C NMR (150 MHz, $CDCl_3$): δ 169.42, 159.08, 144.72, 135.68, 135.61, 135.18, 130.83, 130.39, 130.35, 129.49, 128.41, 127.44, 117.94, 113.69, 109.84, 87.0, 61.47, 61.38, 58.77, 58.72, 58.64, 58.59, 55.64, 43.67, 43.59, 25.09, 25.04, 24.98, 24.93, 20.78, 20.74, 20.69, 12.82. *complicated spectrum due to presence of two diastereomers and overlapping signals.^{31}P NMR (243 MHz, $CDCl_3$): δ 150.62, 150.35. ESI-HRMS (+ve): expected 807.3517, found 807.3530 (M+H, 80%); expected 829.3337, found 829.3348 (M+Na, 60%).

***tert*-Butyl 6-(*N*-(*tert*-butoxycarbonyl)benzamido)-9*H*-purine-9-carboxylate (15):** Boc_2O (2.37 g, 10.87 mmol) was added to a stirred suspension of N^6-benzoyl adenine (1.0 g, 4.18 mmol) and DMAP (102.6 mg, 0.84 mmol) in THF (15 mL). The reaction mixture was stirred overnight at room temperature. The volatiles were then removed under reduced pressure and the residue was purified by FC (SiO_2) with eluent mixture *n*-hexane: EtOAc (85:15) to afford 1.43 g (78%) of **15** as syrup. TLC (SiO_2), *n*-hexane: EtOAc (1:1): R_f 0.70. ^{1}H NMR (600 MHz, DMSO-d_6): δ 8.98 (s, 1H, H(2)), 8.93 (s, 1H, (C8)H), 7.76 (d, J = 8.3, 2H, Bz), 7.69 – 7.65 (m, 1H, Bz), 7.55 (t, J = 7.8, 2H, Bz) 1.66 (s, 9H, N^9 Boc CH_3), 1.28 (s, 9H, N^6 Boc CH_3).

***tert*-Butyl benzoyl(9*H*-purin-6-yl)carbamate** (**16**)**:** A saturated solution of aq. $NaHCO_3$ (17.5 mL) was added to a stirred solution of **15** (1.43 g, 3.26 mmol) in MeOH (35 mL) and the reaction mixture became cloudy. The suspension was then warmed to 50 °C for 1h. When the conversion was quantitative according to TLC, MeOH was removed under reduced pressure. Water (35 mL) was added and the aqueous layer was extracted three times with $CHCl_3$. The organic layer was dried over sodium sulfate. Solvent was removed under reduced pressure (rotovap) and the residue was subjected to purification by FC (SiO_2) with eluent mixture *n*-hexane: EtOAc (1:1) to afford 887 mg (80%) of **16** as foamy solid. TLC (SiO_2), EtOAc: R_f 0.54.^{1}H NMR (600 MHz, DMSO-d_6): δ 8.80 (s, 1H, H(C2)), 8.65 – 8.60 (br s, 1H, C(8)H), 7.76 (d, J = 7.0, 2H, Bz), 7.67 – 7.63 (m, 1H, Bz), 7.54 (t, J = 7.8, 2H, Bz), 1.26 (s, 9H, N^6 Boc CH_3).

(*S*)-*N*-(1-(1-(Bis(4-methoxyphenyl)(phenyl)methoxy)-3-((*tert*-butyldiphenylsilyl)oxy)propan-2-yl)-2-oxo-1,2-dihydropyrimidin-4-yl)isobutyramide (**20**)**:** To a heterogeneous solution of the alcohol compound **10** (1.62 g, 2.6 mmol), N^4-isobutyryl cytosine (520 mg, 2.8 mmol), and triphenylphosphine (820 mg, 3.1 mmol) in anhydrous 1,4-dioxane (25 mL) was added drop wise (~ 5 min) diisopropyl azodicarboxylate (DIAD) (630 µL, 3.1 mmol) at room temperature under N_2 atmosphere. During the dropping DIAD the reaction mixture was turned into a heterogeneous yellow solution. Once DIAD was added completely, the reaction mixture was immediately subjected to sonication for about 5 minutes, at which point the reaction mixture was a clean yellow solution. The reaction mixture was stirred for 1 hour at room temperature when no further change was observed by TLC analysis. The reaction mixture was concentrated in vacuo and purified by silica-gel column chromatography (column dimensions ID, 4 cm x length, 45 cm; flow rate, controlled by pressure; loading silica gel, 120 g) using *n*-hexane:EtOAc (7:3 including 1% TEA) as eluent to afford 1.41 (68%) of **20** as white foam. TLC (SiO_2), R_f = 0.8, *n*-hexane:EtOAc = 7:3. ^{1}H NMR (600 MHz, $CDCl_3$): δ 8.49 (b s, 1H, NH). 7.37 – 6.77 (m, 25H, ArH, C(5,6)H), 5.11 (br s, 1H, CH), 3.96 (J = 4.8 Hz, 2H, CH_2), 3.78 (2s, 6H, OCH_3), 3.55 (d, J = 6.0 Hz, 2H, CH_2), 2.70 – 2.61 (m, 1H, CH), 1.25 (d, J = 1.2 Hz, 3H, $C(CH_3)$), 1.24 (d, J = 1.2 Hz, 3H, $C(CH_3)$), 0.94 (s, 9H, ^{t}Bu). ^{13}C NMR (150 MHz, $CDCl_3$): 177.27 (CO), 161.96 (CO), 159.01, 156.42, 148.04, 144.66, 135.79 – 113.64, 95.64, 86.89 (C-DMTr), 64.74 (CH_2), 62.46 (CH_2), 61.01 (CH),

55.60 (OCH_3), 37.12 (CH), 27.12 (CH_3), 19.48 (C-^{t}Bu), 19.46 (CH_3). ESI-MS (+ve): 279.1 (100%), 301.1 (30%), 342.1 (30%), 557.2 (50%), 579.2 (90%), 796.4 (M+H, 30%), 857.2 (M+Na, 40%).

(*R*)-*N*-(1-(1-(Bis(4-methoxyphenyl)(phenyl)methoxy)-3-hydroxypropan-2-yl)-2-oxo-1,2-dihydropyrimidin-4-yl)isobutyramide (**21**)**:** To a solution of the N^4-^{i}Bu-cytosine derivative **20** (401 mg, 0.5 mmol) in anhydrous THF (25 mL) was added slowly drop wise (~2 minutes) 1M tetrabutylammonium fluoride in THF (2.0 mL) at 1 °C (temperature was measured in ice-bath system) under N_2 atmosphere. After removing the ice-bath, the colorless clean reaction mixture was allowed to warm to room temperature and vigorously stirred for 1 hour, at which point starting material was completely consumed and a new single spot was observed by TCL monitoring. CH_2Cl_2 (20 mL) was added into the reaction mixture. The organic layer was washed with H_2O (20 mL) and brine (20 mL), and then dried over Na_2SO_4. The filtrate was concentrated in vacuo. The yellow foam residue was purified by silica gel column chromatography (column dimensions ID, 3 cm x length, 30 cm; flow rate, controlled by pressure; loading silica gel, 50 g) using *n*-hexane:EtOAc (4:1 including 1% TEA) as eluent to afford 218 mg (78%) of **21** as white foam. TLC (SiO_2), R_f = 0.63, *n*-hexane:EtOAc = 4:1. ^{1}H NMR (600 MHz, $CDCl_3$): δ 7.76, 7.45 – 7.19, 6.83 (m, 15H, ArH, C(5,6)H), 4.85 – 4.78 (br m, 1H, CH), 4.00 (dd, J = 12.0, 6.0 Hz, 2H, CH_2), 3.65 (dd, J = 12.0, 6.0 Hz, 1H, H of CH_2), 3.56 (dd, J = 12.0, 6.0 Hz, 1H, H of CH_2), 2.70 – 2.60 (m, 1H, CH), 1.30 – 1.20 (2d overlapping, J = 1.2 Hz, 6H, $C(CH_3)_2$). ^{13}C NMR (150 MHz,

$CDCl_3$): δ 177.49 (CO), 162.51 (CO), 159.57, 157.26, 149.30 (CH), 144.99, 136.31, 130.67, 128.88, 128.72, 127.91, 114.12 (CH), 96.83 (CH), 87.57 (C), 62.97 (CH_2), 62.00 (CH_2), CH not seen, 56.03 (OCH_3), 37.81 (CH), 19.87 (CH_3). ESI-MS (+ve): 558.3 (M+H, 100%), 580.2 (M+Na, 5%).

(*S*)-3-(Bis(4-methoxyphenyl)(phenyl)methoxy)-2-(4-isobutyramido-2-oxopyrimidin-1(2*H*)-yl)propyl (2-cyanoethyl) diisopropylphosphoramidite (**22**)**:** A solution of the 3'-OH-cytosine derivative **21** (218 mg, 0.4 mmol) and diisopropylethylamine (0.4 mL, 2.3 mmol) in anhydrous CH_2Cl_2 (12 mL) was added drop wise 2-cyanoethyl *N*,*N*-diisopropylchlorophosphoramidite (120 µL, 0.5 mmol) at 0 °C under N_2 atmosphere. The yellowish clean reaction mixture was allowed to warm to room temperature and stirred for 1.5 hour, at which point starting material was remained based on TLC monitoring. An additional 2-cyanoethyl *N*,*N*-diisopropylchlorophosphoramidite (50 µL, 0.2 mmol) was added to complete the reaction. After 30 minutes, no further improvement was observed in the product formation by TLC analysis. The reaction mixture was washed with aqueous, saturated $NaHCO_3$ (20 mL) and the organic layer (CH_2Cl_2) was dried over Na_2SO_4. The filtrate was concentrated in vacuo. The yellow foam was purified by silica gel column chromatography (column dimensions ID, 3 cm x length, 30 cm; flow rate, controlled by pressure; loading silica gel, 80 g) using CH_2Cl_2:MeOH (20:1 including 1% TEA) as eluent to afford 169 mg of **22** (54%) as white foam. TLC (SiO_2), R_f = 0.6, CH_2Cl_2:CH_3OH = 10:1. ^1H NMR (600 MHz, $CDCl_3$): δ 8.49 (br s, 1H, NH), 7.51 – 6.81 (m, 15H, ArH), 5.13 (br s, 1H, CH),

4.30 – 3.84 (m, 3H, CH, CH_2), 3.80 (2s, 6H, OCH_3), 3.76 – 3.62 (m, 2H, CH_2), 3.60 – 3.42 (m, 3H, CH, CH_2) 2.82 – 2.55 (m, 3H, CH and CH_2), 1.32 – 1.02 (d, J = 3.0 Hz, 18H, CH_3). ^{13}C NMR (150 MHz, $CDCl_3$) δ 159.00 (CO), 144.60, 137.68, 135.67, 135.59, 130.26, 128.38, 128.31, 127.35, 118.90, 117.29, 113.67, 105.61, 96.85, 86.95 (C-DMTr), 77.24, 64.73 (CH), 61.64 (CH_2), 60.17 (CH_2), 58.93, 58.59, 58.30, 55.60, 53.84, 46.92, 46.38, 45.71, 36.66, 23.99, 23.26, 23.25, 22.66, 20.49, 19.91, 19.57, 19.53. *complicated spectrum due to a mixture of diastereomers and overlapping signals. ^{31}P NMR (243 MHz, $CDCl_3$): δ 150.78. ESI-HRMS (+ve): 320.25 (100%), 445.36 (30%), 558.26 (20%), expected 758.3667, found 758.3669 (M+H, 10%).

(*S*)-9-(1-(Bis(4-methoxyphenyl)(phenyl)methoxy)-3-((*tert*-butyldiphenylsilyl)oxy)propan-2-yl)-2-isobutyramido-9*H*-purin-6-yl diphenylcarbamate (**23**)**:** To a heterogeneous solution of the alcohol compound **10** (1.71 g, 2.7 mmol), $N^{\mathbf{2}}$-(isobutyryl-O^6-diphenylcarbamoyl)guanine (1.21 g, 3.2 mmol), and triphenylphosphine (1.38 g, 5.2 mmol) in anhydrous 1,4-dioxane (30 mL) was added drop wise (~ 5 minutes) diisopropyl azodicarboxylate (DIAD) (1.1 mL, 5.4 mmol) at room temperature under N_2 atmosphere. During dropping the DIAD, the reaction mixture was turned into a heterogeneous yellow solution. Once DIAD was added completely, the reaction mixture was allowed to warm to 65 ^oC and stirred for 6 hours, at which point the reaction mixture was almost homogeneous yellow solution and no starting material was observed by TLC monitoring. The reaction mixture was allowed to cool to room temperature and

concentrated in vacuo. The dark brown gum was purified by silica-gel column chromatography (column dimensions ID, 3 cm x length, 30 cm; flow rate, controlled by gravity; loading silica gel, 80 g) using *n*-hexane:EtOAc (7:3 including 1% TEA) as eluent to afford 1.28 g (47%) of **23** as white foam. TLC (SiO_2), R_f = 0.7, *n*-hexane:EtOAc = 7:3. ^{1}H NMR (600 MHz, $CDCl_3$): δ 8.18 (s, 1H, C(8)H), 7.72 – 6.72 (m, 33H, ArH,), 5.68 – 5.58 (m, 1H, CH), 4.15 – 4.04 (dd, *J* = 11.4, 5.4 Hz, 2H, CH_2), 3.75 (s, 6H, OCH_3), 3.65 – 3.53 (dd, *J* = 9.6, 5.4 Hz, 2H, CH_2), 3.37 (br m, 1H, CH), 1.25 – 1.22 (2d, *J* = 1.2 Hz, 6H, $C(CH_3)_2$), 0.96 (s, 9H, $C(CH_3)_3$). ^{13}C NMR (150 MHz, $CDCl_3$): δ 163.36 CO), 158.84 (CO), 157.39, 153.08, 148.84, 145.89, 145.50, 141.91, 136.18 – 126.38 (ArH), 113.48, 108.22, 86.70 (C-DMTr), 62.65 (CH_2), 61.76 (CH_2), 60.78 (CH), 55.56 (OCH_3), 27.15 (C), 19.58 (CH_3), 14.61 (CH_3). ESI-MS (+ve): 279.09 (95%), 557.18 (100%), 857.25 (70%), 1031.45 (M+H, 25%).

(*R*)-9-(1-(Bis(4-methoxyphenyl)(phenyl)methoxy)-3-hydroxypropan-2-yl)-2-isobutyramido-9*H*-purin-6-yl diphenylcarbamate (**24**)**:** To a solution of the guanine derivative **23** (824 mg, 0.8 mmol) in anhydrous THF (35 mL) was added slowly drop wise (~ 2 minutes) 33% triethylamine.hydrogenfluoride (2.2 mL) at room temperature under N_2 atmosphere. The slightly yellow reaction mixture was stirred at room temperature for 22 hours, at which point no more starting material observed by TLC monitoring. The reaction mixture was concentrated in vacuo. The residue was dissolved in ethyl acetate (20 mL) and washed with aqueous, saturated $NaHCO_3$ (10 mL) and brine (10 mL). The organic layer was dried over

Na_2SO_4 and the filtrate was concentrated in vacuo. The yellow sticky oil was purified by silica gel column chromatography (column dimensions ID, 3 cm x length, 30 cm; flow rate, controlled by pressure; loading silica gel, 80 g) using CH_2Cl_2:CH_3OH (50:1 including 2 % TEA) as eluent to afford 477 mg of **24** (73 %) as white foam. TLC (SiO_2), R_f = 0.55, CH_2Cl_2:CH_3OH = 40:1. ^{1}H NMR (600 MHz, $CDCl_3$): δ 8.21, 8.05 (2s, 1H, NH, C(8)H), 7.63 – 6.77 (m, 24H, ArH), 5.95 - 5.75 (br s, 1H, OH), 4.49 – 4.41 (m, 1H, CH), 4.15 – 4.10 (br m, 2H, CH_2), 3.85 – 3.75 (m, 2H, CH_2), 3.75 – 3.70 (2s, 6H, OCH_3), 3.57 (m, 2H, CH_2), 2.55 – 2.65 (m, 1H, CH), 1.28 – 1.23 (2d, J = 6.0 Hz, 6H, $C(CH_3)_2$). ^{13}C NMR (150 MHz, $CDCl_3$): δ 163.77 (CO), 159.21(CO), 157.78, 153.46, 149.33, 146.06, 145.50, 142.27, 136.39, 133.73 – 126.78 (Ar-C), 113.38, 108.87, 87.06 (C-DMTr), 65.27 (CH_2), 62.90 (CH_2), 61.99 (CH), 56.04 (OCH_3), 27.56 (CH), 20.05, 19.94 (CH_3). ESI-HRMS (+ve): 303.14 (30%), 765.30 (100%), Expected 793.3344 (M+H); found 793.3348 (M+H, 10%).

9-((2*S*)-1-(Bis(4-methoxyphenyl)(phenyl)methoxy)-3-(((2-cyanoethoxy)(diisopropylamino)phosphino)oxy)propan-2-yl)-2-isobutyramido-9*H*-purin-6-yl diphenylcarbamate (**25**)**:** A solution of the 3'-OH-guanine derivative **24** (230 mg, 0.4 mmol) and diisopropylethylamine (0.4 mL, 2.3 mmol) in anhydrous CH_2Cl_2 (15 mL) was added drop wise 2-cyanoethyl *N*,*N*-diisopropylchlorophosphoramidite (150 μL, 0.6 mmol) at 0 °C under N_2 atmosphere. The pale brown reaction mixture was allowed to warm to room temperature and stirred for 1.5 hour, at which point starting material was

remained based on TLC monitoring. An additional 2-cyanoethyl *N*,*N*-diisopropylchlorophosphoramidite (75 μL, 0.3 mmol) was added to complete the reaction. After 30 minutes, no further improvement was observed in the product formation by TLC analysis. The reaction mixture was washed with aqueous, saturated $NaHCO_3$ (20 mL) and the organic layer (CH_2Cl_2) was dried over Na_2SO_4. The filtrate was concentrated in vacuo. The yellow foamy residue was purified by silica gel column chromatography (column dimensions ID, 3 cm x length, 30 cm; flow rate, controlled by pressure; loading silica gel, 50 g) using CH_2Cl_2:MeOH (20:1 including 1% TEA) as eluent to afford 148 mg of **25** (51 %) as white foam. TLC (SiO_2), R_f = 0.57, CH_2Cl_2:CH_3OH = 20:1. ^{1}H NMR (600 MHz, CD_3OD): δ 8.31 (d, J = 6.0 Hz, 1H, NH), 7.55 – 6.60 (m, 25H), 5.12 – 4.95 (m, 1H, CH), 4.34 – 4.01 (m, 2H, CH_2), 3.77 – 3.25 (m, 7H, CH, CH_2), 3.65 – 3.62 (2 s, 6H, CH_3), 2.59 – 2.48 (m, 2H, CH_2), 1.07, 1.06, 0.99, 0.90 (4d, J = 12.0, 18H). ^{13}C NMR (150 MHz, CD_3OD): δ 171.84 (C), 159.50, 159.45, 156.30, 155.81, 155.75, 152.84. 152.81, 151.60, 151.58, 146.15, 146.06, 145.48, 142.73, 136.07, 135.81, 135.77, 130.58, 130.43, 130.41, 129.75, 128.34, 128.28, 127.26, 121.03, 117.93, 113.52, 87.11, 62.96, 62.46, 61.93 (d, J_{CP} = 15 Hz), 59.09(d, J_{CP} = 4.5 Hz), 58.95 (d, J_{CP} = 4.5 Hz), 55.71, 43.79, 43.71, 24.52, 24.47, 24.46, 24.41, 24.38, 24.37, 20.35, 20.31, 20.26. *complicated spectrum. Many peaks doubled due to its diastereomeric relationship and overlapping signals. ^{31}P NMR (243 MHz, CD_3OD): δ 149.54, 149.46. ESI-MS (+ve): 303.14 (100%), 746.22 (10%), 825.36 (5%), 994.34 (M+H, 1%).

Synthesis of 26: Method I

(*S*)-*N*-(9-(1-(Bis(4-methoxyphenyl)(phenyl)methoxy)-3-((*tert*-butyldiphenylsilyl)oxy)propan-2-yl)-9*H*-purin-6-yl)benzamide (**26**): To a white suspension of the alcohol compound **10** (638 mg, 1.0 mmol), N^6-benzoyl adenine (478 mg, 2.0 mmol), and triphenylphosphine (PPh_3) (502 mg, 2.0 mmol) in anhydrous 1,4-dioxane (20 mL) was added diisopropyl azodicarboxylate (DIAD) (405 mg, 2.0 mmol) slowly drop-wise (~ 10 minutes) at room temperature under N_2 atmosphere. The white heterogeneous reaction mixture was slowly turned into yellowish clean solution as the reaction mixture was subjected to sonication. The reaction was stopped when no further improvement was observed in the product formation (~ 1 hour). The reaction mixture was concentrated in vacuo. And, the yellow gum residue was tried to be purified by silica-gel column chromatography (column dimensions ID, 3 cm x length, 30 cm; flow rate, controlled by gravity; loading silica gel, 70 g) using *n*-hexane:EtOAc (3:2 including 1% NEt_3) as eluent to afford 297 mg (34 %) of **26** as white foam. TLC (SiO_2), R_f = 0.67, *n*-hexane:EtOAc = 3:2 (No TEA). ^{1}H NMR (600 MHz, $CDCl_3$): δ 9.01 (s, 1H, NH), 8.71, 8.05 (2s, 1H each, C(8)H, C(2)H), 8.04 (br m, 2H, ArH), 7.65 – 6.72 (m, 23H), 4.99 – 4.90 (br m, 1H, CH), 4.20 – 4.04 (m, 2H), 3.76, 3.75 (2s, 6H, OCH_3), 3.81 – 3.73 (m, 1H, hidden under OCH_3 peak, H of CH_2), 3.61 (dd, J = 12.0, 6.0 Hz, 1H), 0.92 (s, 9H, CH_3). ^{13}C NMR (150 MHz, $CDCl_3$): δ 165.03 (CO), 158.97, 152.60, 152.51, 149.66, 149.66, 144.63, 143.32, 136.34, 135.88, 135.77, 135.66, 134.27, 133.08, 132.78, 132.63, 130.40 – 130.24, 129.24, 128.27 – 128.19, 127.33, 123.12, 113.60, 87.02 (C-DMTr), 62.82

(CH_2), 61.78 (CH_2), 57.70 (CH), 55.62 (OCH_3), 27.13 (C), 19.45 (CH_3). ESI-MS (+ve): 854 (M+H, 100%), 876 (M+Na, 5%).

<u>Synthesis of 26: Method II</u>

(*S*)- Di-*tert*-butyl 9-(1-(bis(4-methoxyphenyl)(phenyl)methoxy)-3-((*tert*-butyldiphenylsilyl)oxy)propan-2-yl)-9*H*-purin-6-yl)iminodcarboxylate (**29**)**:** To a solution of the alcohol derivative **10** (1.26 g, 2.0 mmol), triphenyphosphine (1.06 g, 4.0 mmol) and N^6-di Boc-adenine (1.34 g, 4.0 mmol) in anhydrous 1,4-dioxane (20 mL) were added slowly drop wise (~15 minutes) diisopropyl azodicarboxylate (DIAD) (0.8 mL, 4.0 mmol) at room temperature under N_2 atmosphere. When DIAD was added about 60% of the amount, the homogenous clean reaction solution was turned into dark yellow solution. After completing addition of DIAD, the reaction mixture was turned into bright yellow heterogeneous solution. After stirring additional 1 hour, at which point no more starting material was observed by TLC monitoring, the reaction mixture was concentrated in vacuo. The dark yellow gem was purified by silica gel column chromatography (column dimensions ID, 3 cm x length, 30 cm; flow rate, controlled by gravity; loading silica gel, 80 g) using *n*-hexane:EtOAc (3:1 including 1% NEt_3) as eluent to afford 1.40 g (74 %) of **29** as white foam. TLC (SiO_2), R_f = 0.6 (Ethyl acetate: *n*-hexane = 1:3). ^{1}H NMR (600 MHz, $CDCl_3$) δ 8.78, 8.17 (s, 2H, C(8)H, C(2)H), 7.51 – 6.69 (m, 23H, ArH), 4.99 – 4.92 (br s, 1H, CH), 4.15 (dd, J = 10.8, 6.0 Hz, 1H, CH_2), 4.06 (dd, *J* = 10.8, 4.8 Hz, 1H, CH_2), 3.75 (s, 6H, OCH_3), 3.78 – 3.70 (m, 1H, CH_2), 3.55 (dd, *J* = 10.2, 4.8 Hz,

1H, CH_2), 1.41 (s, 18H, CH_3(^{t}Boc)), 0.90 (s, 9H, CH_3(^{t}Bu)). ^{13}C NMR (150 MHz, $CDCl_3$) δ 158.97 (CO), 153.97, 154.05, 152.05, 150.87, 150.55, 145.09, 135.88, 135.78, 135.60, 132.77, 132.63, 130.41, 130.37, 130.36, 130.23, 129.18, 128.30, 128.26, 128.23, 127.29, 113.61, 87.5 (C-DMTr), 83.99 (C), 62.80 (CH_2), 61.92 (CH_2), 57.82 (CH), 55.62 (OCH_3), 28.21 (C), 27.10 (CH_3), 19.44 (CH_3). ESI-MS (+ve): 950.45 (M+H, 75%), 972.43 (M+Na, 25%).

(*S*)-2-(6-Amino-9*H*-purin-9-yl)-3-((*tert*-butyldiphenylsilyl)oxy)propan-1-ol (**33**): To a solution of the di Boc-adenine derivative **29** (2.52 g, 2.6 mmol) in anhydrous CH_2Cl_2 (15 mL) was added slowly drop wise (~ 15 minutes) a 1:1 mixture of trifluoroacetic acid and anhydrous CH_2Cl_2 (total volume, 15 mL) at 0 ^oC under N_2 atmosphere. The reaction mixture was stirred in the ice-bath for 10 min, and then allowed to warm to room temperature. The reaction mixture was vigorous stirred until starting material was completely consumed based on TLC monitoring (~1.5 hour). The reaction mixture was washed with aqueous, saturated $NaHCO_3$ solution (30 mL) and the organic layer was dried over Na_2SO_4. The filtrate was concentrated in vacuo, and the residue was purified by silica gel column chromatography (column dimensions ID, 3 cm x length, 30 cm; flow rate, controlled by pressure; loading silica gel, 50 g) using CH_2Cl_2: MeOH (9:1) as eluent to afford 1.03 g (87 %) of **33** as white foam. TLC (SiO_2), R_f = 0.25 (CH_2Cl_2 : MeOH = 20:1). ^{1}H NMR (600 MHz, DMSO-d_6) δ 8.18, 8.10 (2 s, 2H, C(8)H, C(2)H), 7.47 – 7.16 (m, 12H, ArH, NH_2), 5.18 (m, 1H, OH), 4.75 – 4.68 (m, 1H, CH), 4.13 – 3.88 (m, 4H, CH_2), 0.85 (s, 9H, CH_3). ^{13}C NMR (150 MHz,

DMSO-d_6) δ 156.25, 152.32, 149.92, 141.53, 135.47, 132.67, 129.99, 127.76, 127.73, 119.16, 104.64, 62.65 (CH_2), 60.17 (CH_2), 60.12 (CH), 26.15 (C), 18.86 (CH_3). ESI-MS (+ve): 750.35 (M+H, 95%), 772.33 (M+Na, 5%).

(*S*)-*N*-(9-(1-(Bis(4-methoxyphenyl)(phenyl)methoxy)-3-((*tert*-butyldiphenylsilyl)oxy)propan-2-yl)-9*H*-purin-6-yl)benzamide (**26**): To a solution of the adenine derivative **33** (950 mg, 2.1 mmol) and 4-dimethylamino pyridine (26 mg, 0.2 mmol) in anhydrous pyridine (30 mL) was added 4,4-dimethoxytrityl chloride (DMTrCl) (860 mg, 2.5 mmol) at 0 ^oC under N_2 atmosphere. The reaction mixture was allowed to warm to room temperature. After 3 hour, at which point starting material was still remained based on TLC monitoring, additional DMTrCl (145 mg, 0.4 mmol) was added at room temperature under N_2 atmosphere. After 15 hour, at which point no starting material was observed on TLC analysis, the reaction mixture was directly concentrated in vacuo. The residue was diluted with CH_2Cl_2 (50 mL) and washed with aqueous, saturated $NaHCO_3$ solution (30 mL), H_2O (30 mL), and then brine (30 mL). The organic layer (CH_2Cl_2) was dried over Na_2SO_4 and the filtrate was concentrated in vacuo. The dark yellow gum was purified by silica gel column chromatography (column dimensions ID, 4 cm x length, 45 cm; flow rate, controlled by pressure; loading silica gel, 120 g) using CH_2Cl_2: MeOH (13:1) as eluent to afford 1.19 g (75 %) of 1'-O-DMTr-adenine intermediate **34** as white foam. TLC (SiO_2), R_f = 0.55 (CH_2Cl_2 : MeOH = 9:1). ^1H NMR (600 MHz, CD_3OD) δ 8.58 – 8.54 (m, 2H, NH_2), 8.10, 7.97 (2s, 1H, C(8)H, C(2)H), 7.49 – 6.74 (m,

29H, ArH), 4.95 – 4.85 (m, 1H, CH, hidden under the water peak in CD_3OD), 4.18 (dd, J = 10.8, 6.3 Hz, 1H), 4.11 (dd, J = 10.8, 4.9 Hz, 1H), 3.78, 3.77 (2s, 6H, OCH_3), 3.74 (dd, J = 9.9, 7.4 Hz, 1H), 3.60 (dd, J = 10.0, 4.2 Hz, 1H), 0.91 (s, 9H, CH_3). ^{13}C NMR (150 MHz, CD_3OD) δ 152.38, 149.06, 137.33, 135.56, 135.51, 130.12, 130.09, 130.06, 127.92, 127.83, 127.78, 124.59, 113.09, 113.06 (all ArC), 62.43 (CH_2), CH and CH_2 not visible, 54.67 (OCH_3), 26.18 (C), 18.61 (CH_3).

To a solution of the above 1'-O-DMTr-adenine intermediate **34** (1.02 g, 1.4 mmol) in dry pyridine (20 mL) was added rapidly drop wise benzoyl chloride (190 μL, 1.6 mmol) under ice-bath system (bath temp. -1 to 2 ^oC). After starring in the ice-bath for 10 minutes, the reaction mixture was allowed to warm to room temperature. After 2 hour, at which point no starting material was observed on TLC analysis and the reaction mixture was directly concentrated in vacuo. The residue was diluted with CH_2Cl_2 (100 mL) and washed with aqueous, saturated $NaHCO_3$ solution (50 mL) and H_2O (50 mL). The organic layer (CH_2Cl_2) was dried over Na_2SO_4 and the filtrate was concentrated in vacuo. The dark yellow foam was purified by silica gel column chromatography (column dimensions ID, 3 cm x length, 30 cm; flow rate, controlled by gravity; loading silica gel, 80 g) using CH_2Cl_2:MeOH (30:1 to 20:1 including 1% TEA) as eluent to afford 507 mg (89 %) of **26** as white foam. TLC (SiO_2), R_f = 0.60 (CH_2Cl_2 : MeOH = 30:1). ^1H NMR (600 MHz, $CDCl_3$) δ 9.29 (s, 1H, NH), 8.74, 8.09 (2s, 1H each, C(8)H, C(2)H), 8.11 – 8.04 (br m, 2H, ArH), 7.71 – 6.73(m, 28H, ArH) 4.98 (dq, J = 9.9, 5.3 Hz, 1H),

4.20 (dd, *J* = 10.9, 5.7 Hz, 1H), 4.12 (dd, *J* = 10.9, 4.7 Hz, 1H), 3.83 – 3.76 (2s and m, 7H, OCH_3 and H of CH_2), 3.64 (dd, *J* = 10.0, 4.6 Hz, 1H, H of CH_2), 0.94 (s, 9H, CH_3). ^{13}C NMR (150 MHz, $CDCl_3$) δ 164.64 (CO), 158.79, 152.42, 152.28, 149.35, 144.41, 143.13, 135.67, 135.56, 135.47, 134.07, 132.90, 132.60, 132.45, 130.18, 130.14, 130.07, 130.03, 129.08, 128.10, 128.05, 128.01, 127.98, 127.12, 113.40, 86.82 (C-DMTr), 62.63 (CH_2), 61.58 (CH_2), 57.53 (CH), 55.41(OCH_3), 26.90 (C), 19.23 (CH_3).

(*R*)-*N*-(9-(1-(Bis(4-methoxyphenyl)(phenyl)methoxy)-3-hydroxypropan-2-yl)-9*H*-purin-6-yl)benzamide (**27**): To a solution of the N^6-benzoyl-adenine derivative **26** (400 mg, 0.47 mmol) in anhydrous THF (30 mL) was added slowly drop wise 1M tetrabutylammonium fluoride in THF (2.0 mL) under ice-bath system (-2 °C) and N_2 atmosphere. The reaction mixture was allowed to warm to room temperature. After 25 min, at which point starting material was completely consumed based on TLC analysis, CH_2Cl_2 (20 mL) was added into the reaction mixture. The organic layer was washed with H_2O (20 mL) and brine (20 mL), and then dried over Na_2SO_4. The filtrate was concentrated in vacuo. The yellow foam residue was purified by silica gel column chromatography (column dimensions ID, 3 cm x length, 30 cm; flow rate, controlled by pressure; loading silica gel, 50 g) using CH_2Cl_2: MeOH (10:1 including 1% TEA) as eluent to afford 256 mg (89 %) of **27** as white foam. TLC (SiO_2), R_f = 0.45 (CH_2Cl_2 : MeOH = 10:1).^{1}H NMR (600 MHz, $CDCl_3$) δ 9.47 (br s, 1H, NH), 8.59, 8.10 (s, each 1H, C(8)H, C(2)H), 8.06 (br d, *J* = 6.0 Hz, 2H, ArH), 7.63 – 6.67 (m, 16H, ArH), 4.78 – 4.68 (m, 1H,

2H), 4.29 – 4.05 (m, 2H, CH_2), 3.75, 3.74 (2s, 6H, OCH), 3.79 – 3.61 (m, 2H, CH_2).^{13}C NMR (150 MHz, $CDCl_3$) δ 165.23 (CO), 158.91, 152.12, 151.70, 149.78, 144.55, 144.28, 135.59, 134.02, 133.18, 130.19, 130.16, 129.21, 129.19, 128.37, 128.28, 128.19, 127.33, 123.09, 113.55, 113.52, 87.14 (C-DMTr), 62.51 (CH_2), 62.24, 60.83 (CH), 55.61 (OCH_3). ESI-HRMS (+ve): expected (M+H) 616.2554; found (M+H) 616.2552.

(*S*)-2-(6-Benzamido-9*H*-purin-9-yl)-3-(bis(4-methoxyphenyl)(phenyl)methoxy)propyl (2-cyanoethyl) diisopropylphosphoramidite (**28**)**:** A solution of the N^6-Benzoyl-3'-OH-adenine derivative **27** (340 mg, 0.55 mmol) and diisopropylethylamine (0.7 mL, 3.99 mmol) in anhydrous CH_2Cl_2 (20 mL) was cooled to 0 °C under N_2 atmosphere. 2-Cyanoethyl *N*,*N*-diisopropylchlorophosphoramidite (200 mg, 0.84 mmol) was added rapidly drop wise into the reaction mixture at 0 °C under N_2 atmosphere. The yellowish clean reaction mixture was allowed to warm to room temperature and stirred for 1.5 hour, at which point starting material was remained based on TLC monitoring. An additional 2-cyanoethyl *N*,*N*-diisopropylchloro phosphoramidite (70 µL, 0.28 mmol) was added to complete the reaction. After 30 min, no further improvement was observed in the product formation by TLC analysis. The reaction mixture was washed with aqueous, saturated $NaHCO_3$ (20 mL) and the organic layer (CH_2Cl_2) was dried over Na_2SO_4. The filtrate was concentrated in vacuo. The slightly yellow foam residue was purified by silica gel column chromatography (column dimensions ID, 3 cm x length, 30 cm; flow rate,

controlled by gravity; loading silica gel, 80 g) using CH_2Cl_2:MeOH (20:1 including 1% TEA) as eluent to afford 375 mg (72 %) of **28** as white foam. TLC (SiO_2), R_f = 0.49, CH_2Cl_2: MeOH = 20:1). ^{1}H NMR (600 MHz, CD_3OD): δ 8.66, 8.43, 8.11, 8.09 (4s, 4H, NH, C(8)H, C(2)H), 7.66 – 6.69 (m, 18H, ArH), 5.19 – 5.10 (m, 1H, CH), 4.38 – 4.12 (m, 2H, CH_2), 3.82, 3.71 (2s, 6H, OCH_3), 3.82 – 3.40 (m, 6H, CH, CH_2), 2.75 – 2.67 (m, 1H, CH), 2.65, 2.55 (2t, J = 6.0 Hz, 2H, CH_2), 1.17 – 0.93 (4d, J = 2.9 Hz, 12H, CH_3). ^{13}C NMR (150 MHz, CD_3OD): δ 167.02 (CO), 159.11, 152.82, 151.84, 150.00, 144.87, 144.54, 144.44, 135.59, 135.52, 134.09, 130.12, 130.05, 128.77, 128.43, 127.97, 127.87, 126.90, 123.81, 118.52, 113.17, 86.82, 61.93 (multiplet, CH_2), 58.74 and 58.61 (2d, J = 9Hz , CH_2), 57.85 and 57.55 (d, J = 4 Hz, CH), 54.77 (OCH_3), 43.37, 43.29, 24.10 – 23.92 (multiple peaks), 19.93 – 19.86 (multiple peaks). *complicated spectrum due to a mixture of diastereomers and overlapping signals. ^{31}P NMR (243 MHz, CD_3OD): δ 150.48, 150.03. ESI-MS (+ve): 733 (100%), 747 (75%), 816 (M+H, 45%), 838 (M+Na, 5%).
