# Supplementary material for: Synthesis of phosphoramidites of isoGNA, an isomer of glycerol nucleic acid
Source: Beilstein J Org Chem. 2014 Sep 8;10:2131–8. doi: 10.3762/bjoc.10.220 (PMC4168771; doi:10.3762/bjoc.10.220)
Supplement: File 2 — 1H, 13C and 31P NMR spectra. [file Beilstein_J_Org_Chem-10-2131-s002.pdf]

Supporting Information

for

Synthesis of phosphoramidites of isoGNA, an isomer of glycerol nucleic acid

Keunsoo Kim, Venkateshwarlu Punna, Phaneendrasai Karri and Ramanarayanan Krishnamurthy*

Address: Department of Chemistry, The Scripps Research Institute, 10550 North Torrey Pines Rd, La Jolla, CA 92037, USA

Email: Ramanarayanan Krishnamurthy - rkrishna@scripps.edu

* Corresponding author

^{1}H, ^{13}C and ^{31}P NMR spectra

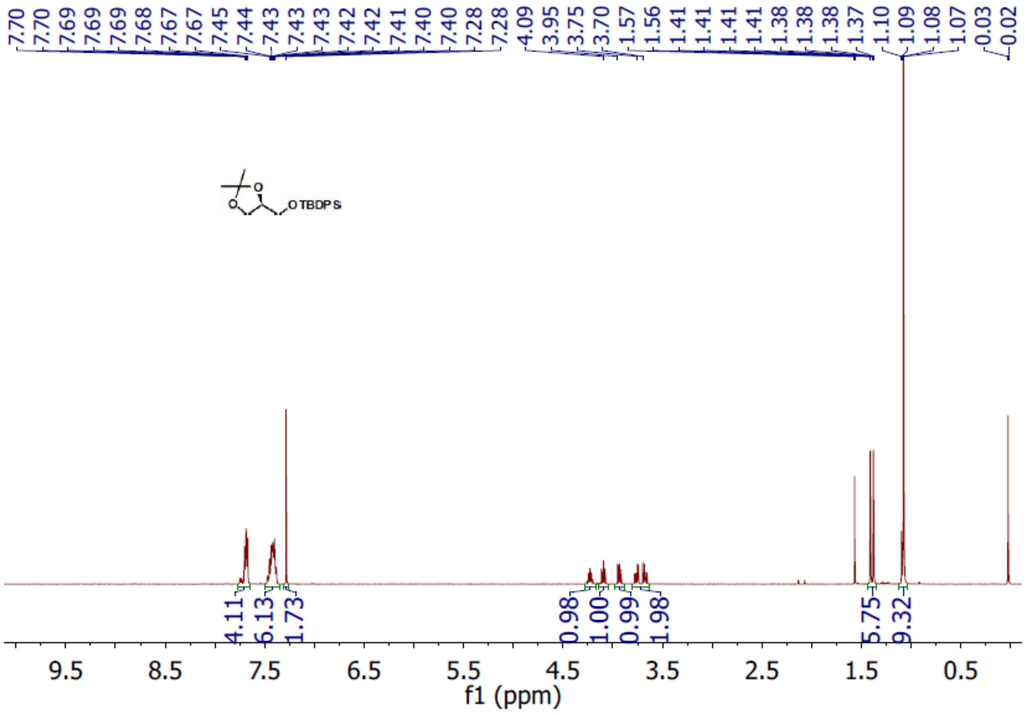

^{1}H NMR spectrum of **8** in $CDCl_3$.

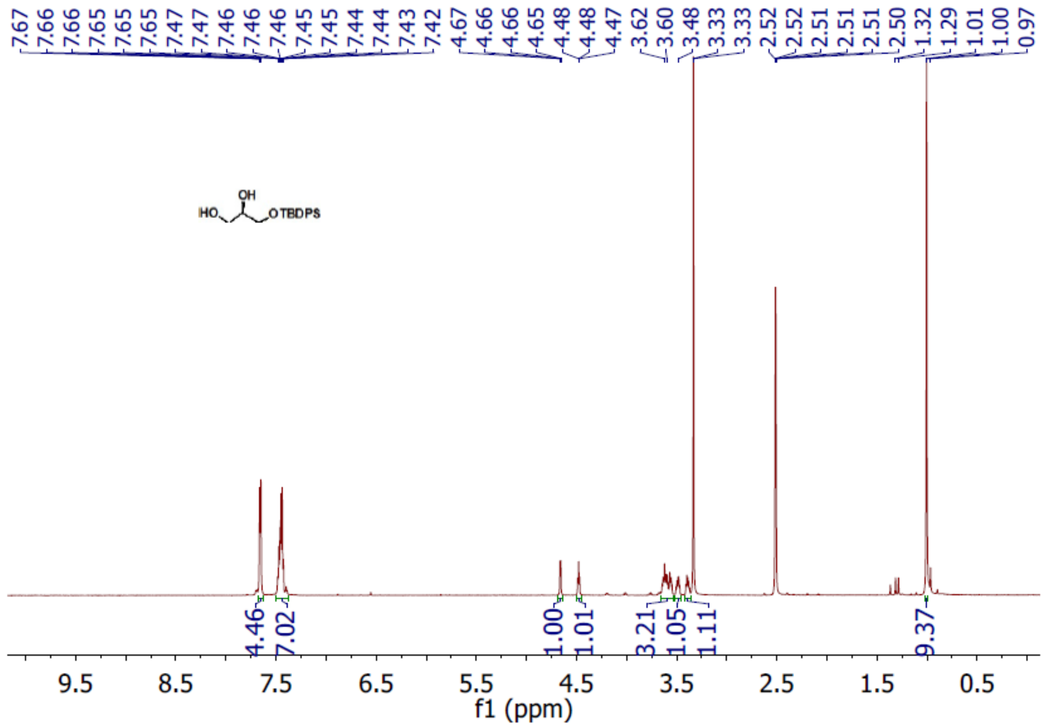

^{1}H NMR spectrum of **9** in DMSO-d_6.

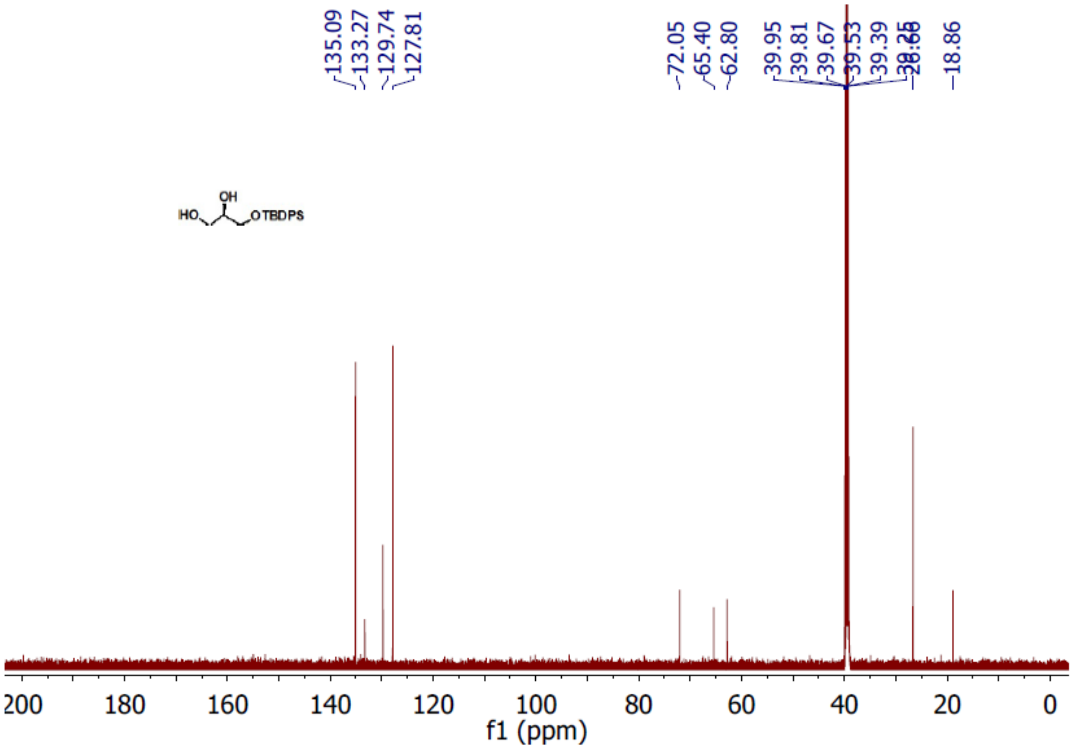

^{13}C NMR spectrum of **9** in DMSO-d_6

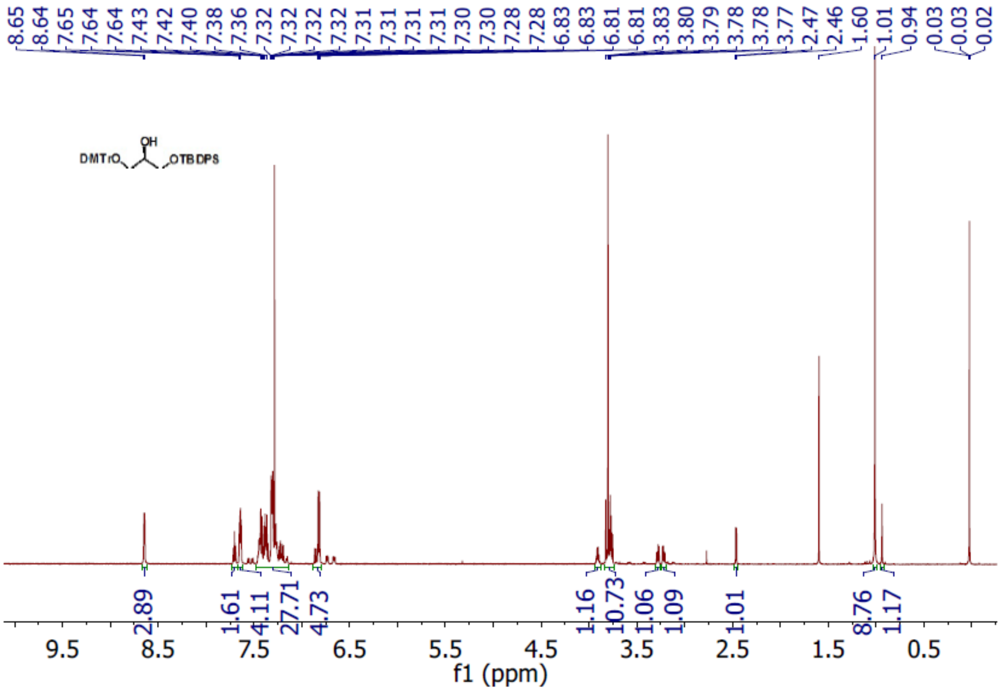

^{1}H NMR spectrum of **10** in $CDCl_3$.

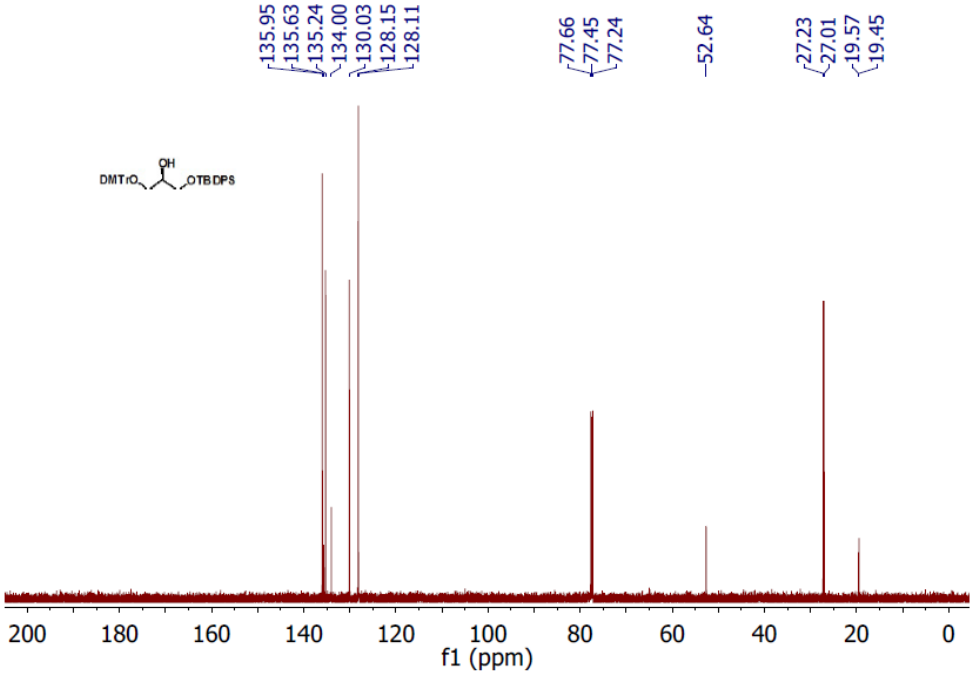

^{13}C NMR spectrum of **10** in $CDCl_3$.

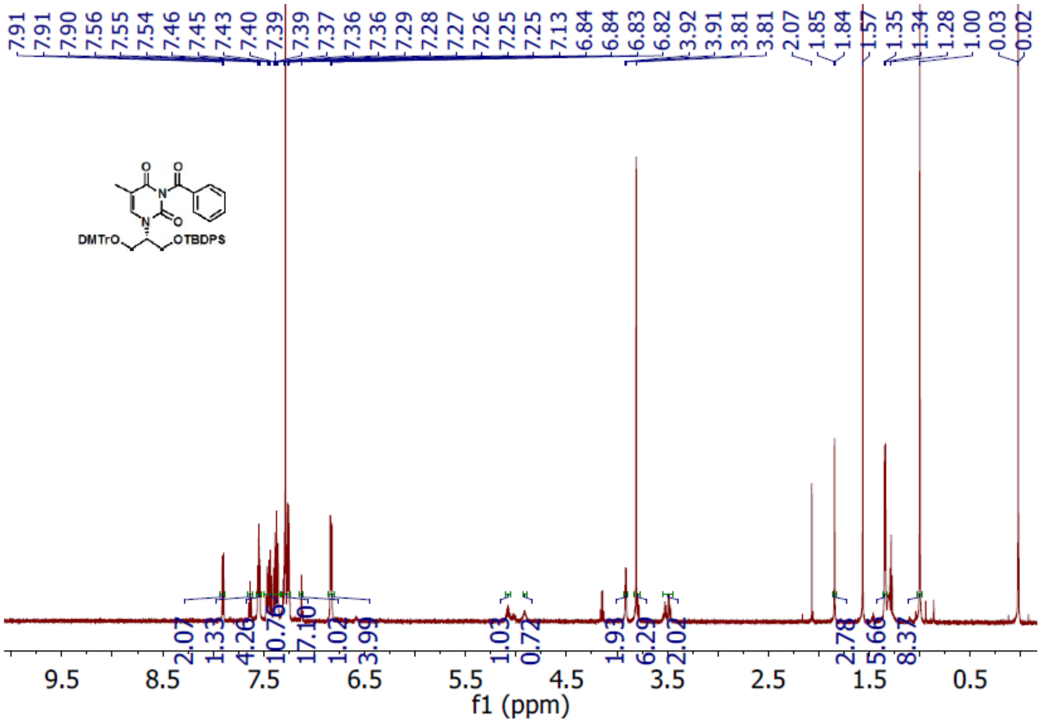

^{1}H NMR spectrum of **11** in $CDCl_3$.

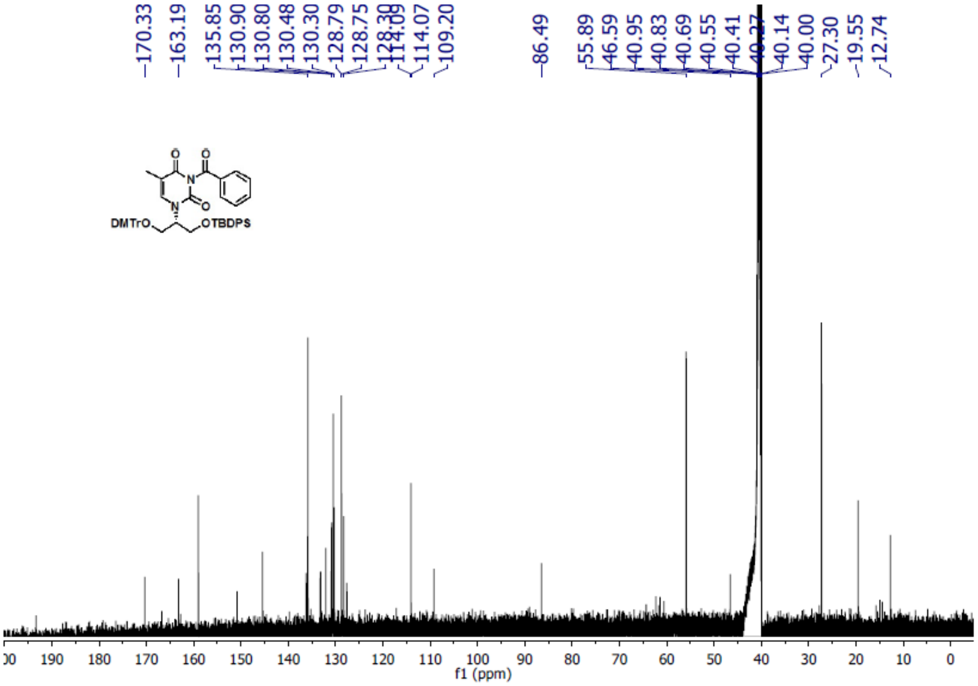

^{13}C NMR spectrum of **11** in DMSO-d_6.

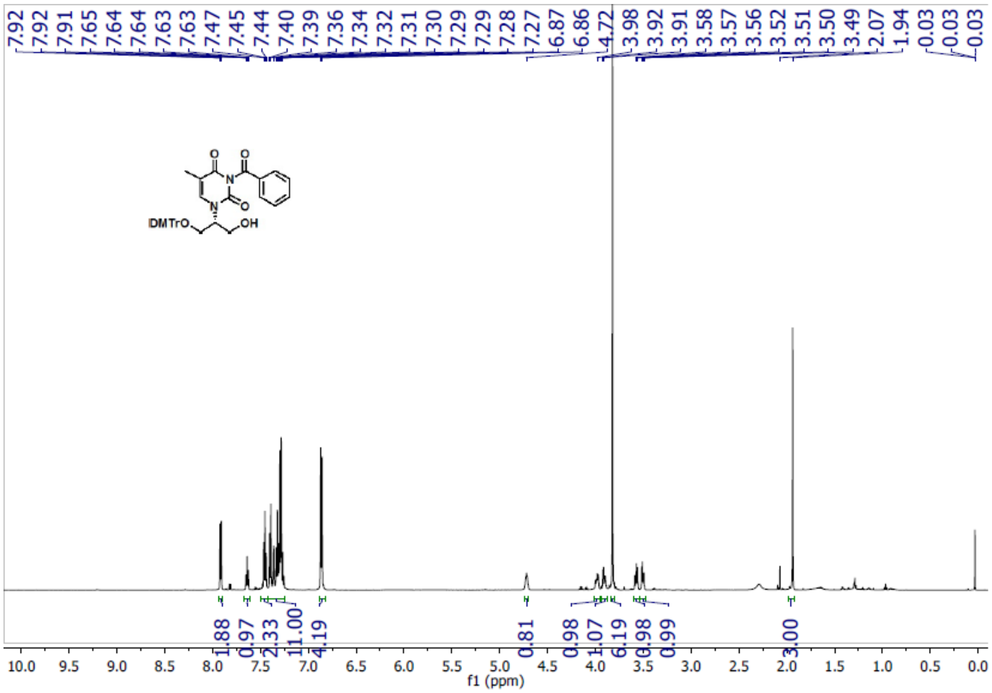

^{1}H NMR spectrum of **12** in $CDCl_3$.

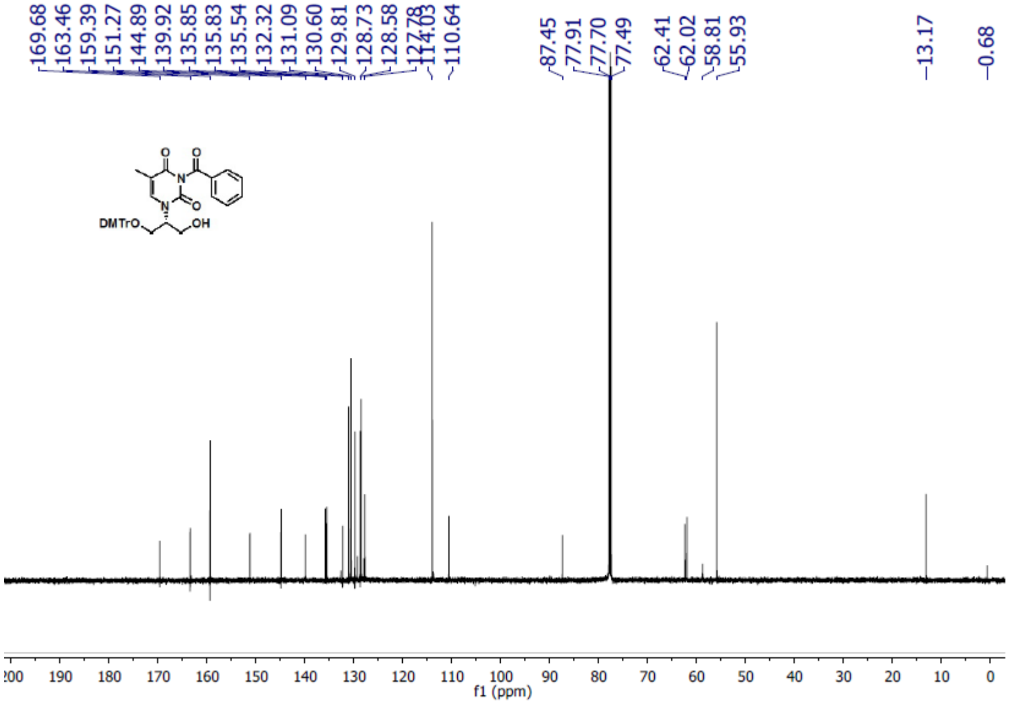

^{13}C NMR spectrum of **12** in $CDCl_3$.

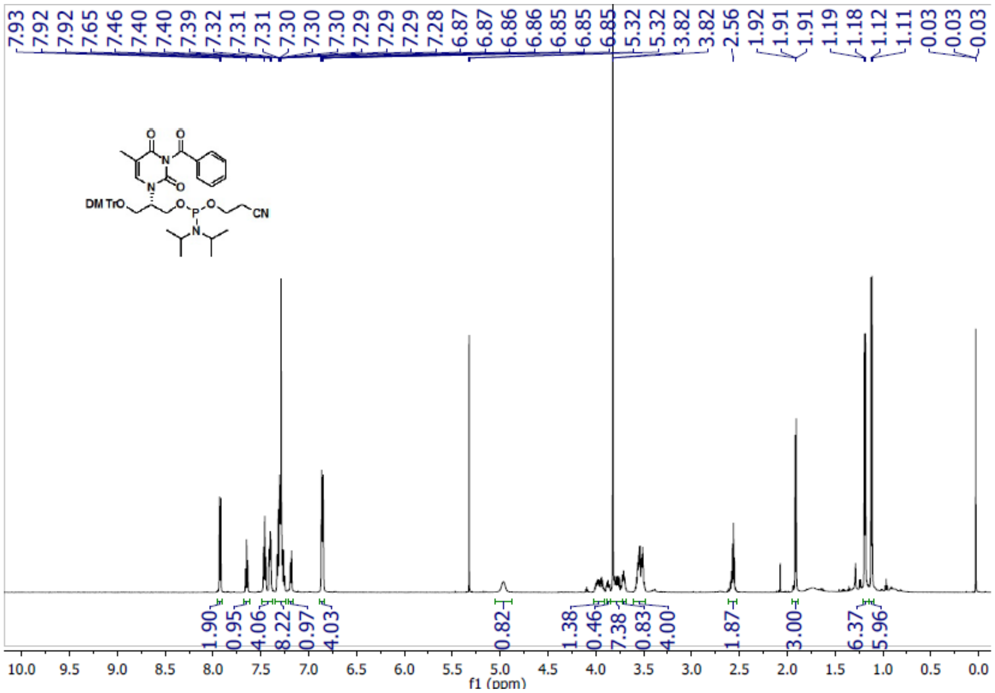

^{1}H NMR spectrum of **13** in $CDCl_3$.

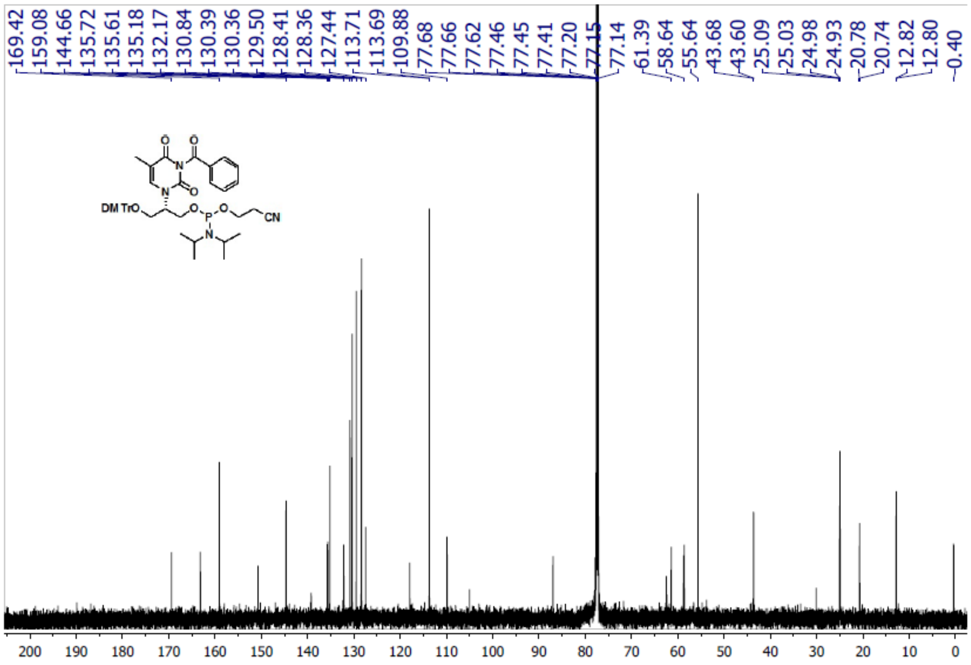

^{13}C NMR spectrum of **13** in $CDCl_3$.

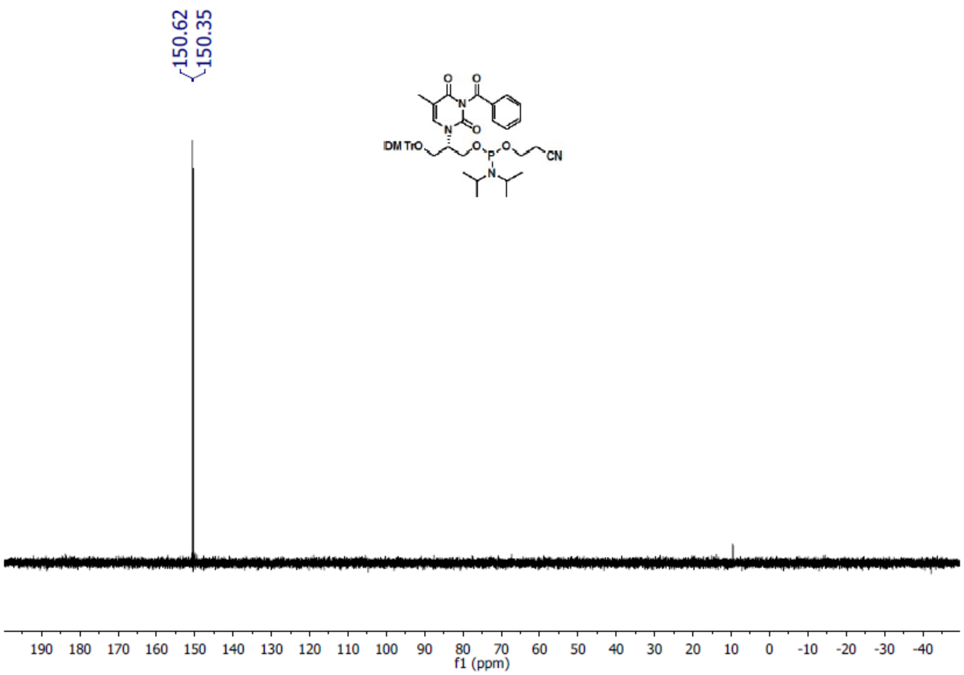

^{31}P NMR spectrum of **13** in $CDCl_3$.

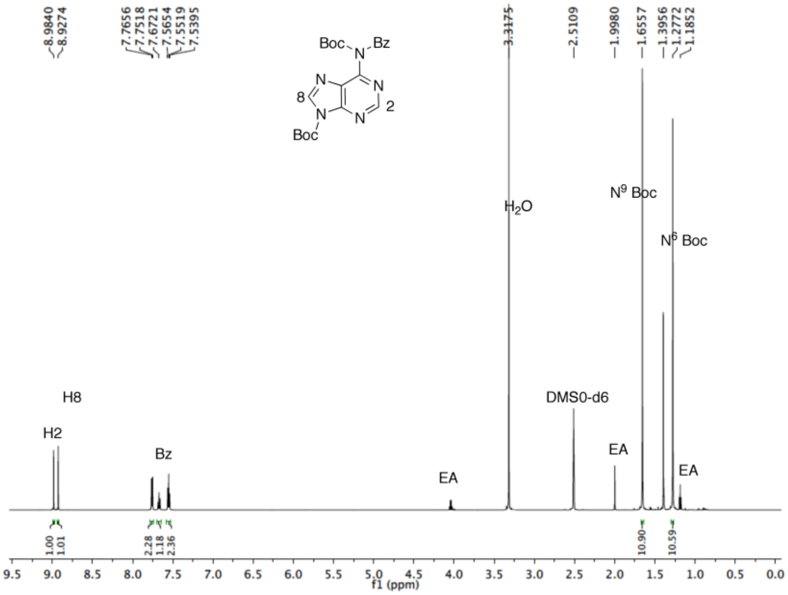

^{1}H NMR spectrum of **15** in DMSO-d_6.

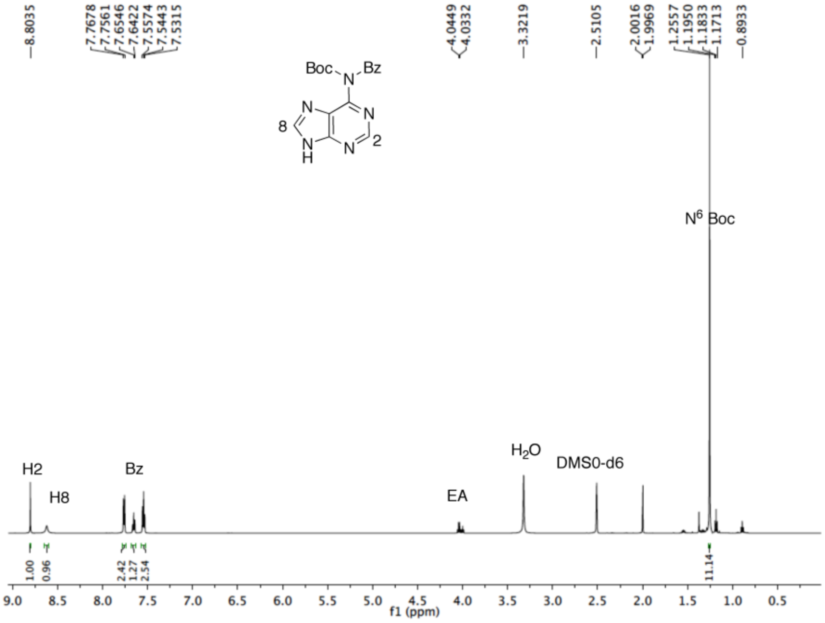

^{1}H NMR spectrum of **16** in DMSO-d_6.

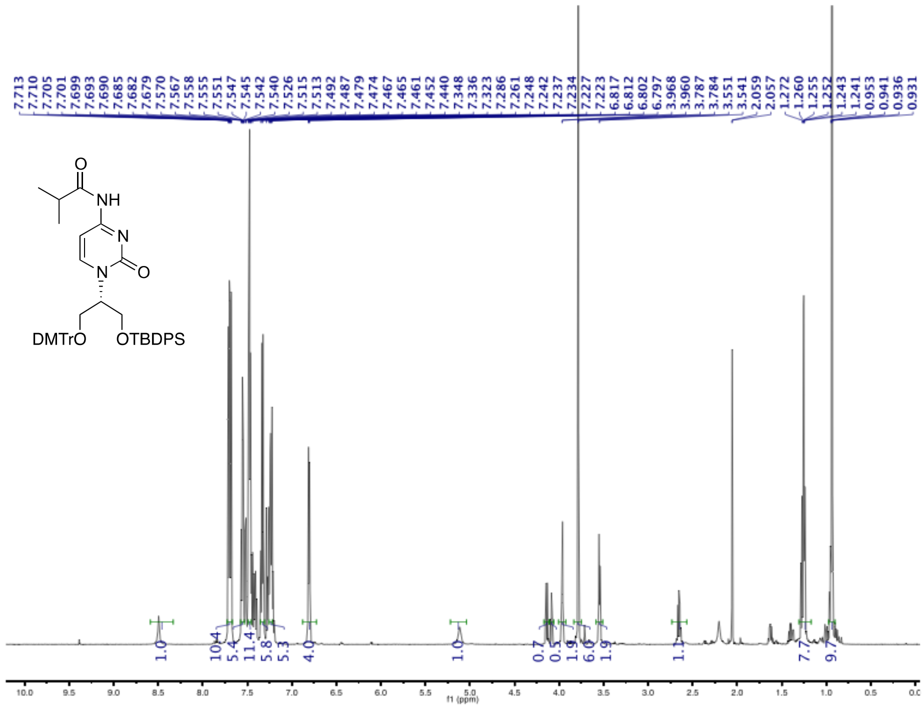

^{1}H NMR spectrum of **20** in $CDCl_3$.

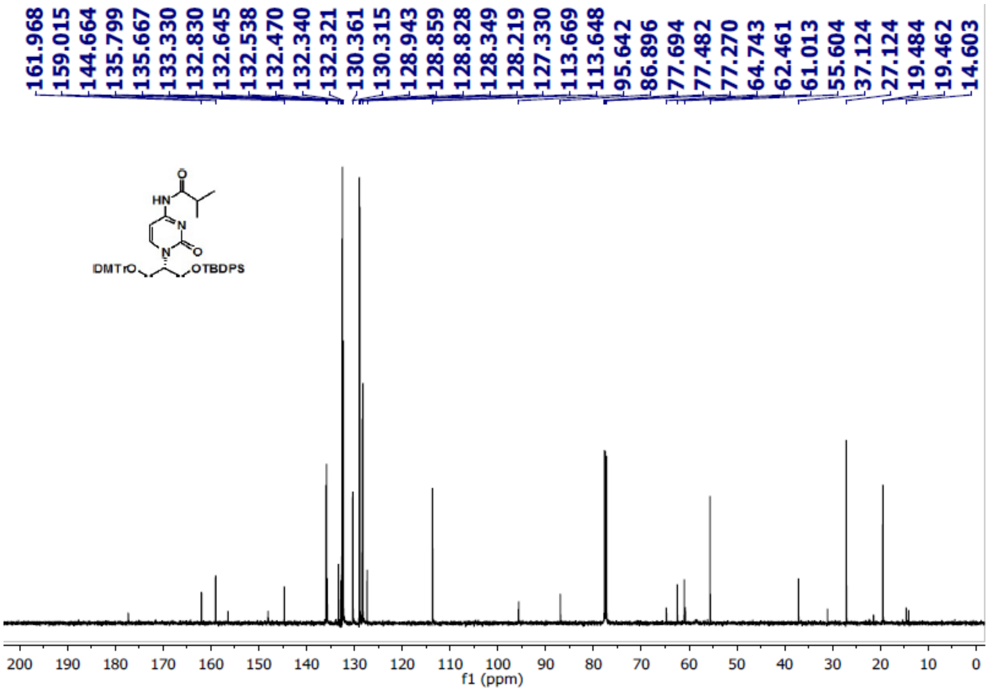

^{1}H NMR spectrum of **20** in $CDCl_3$.

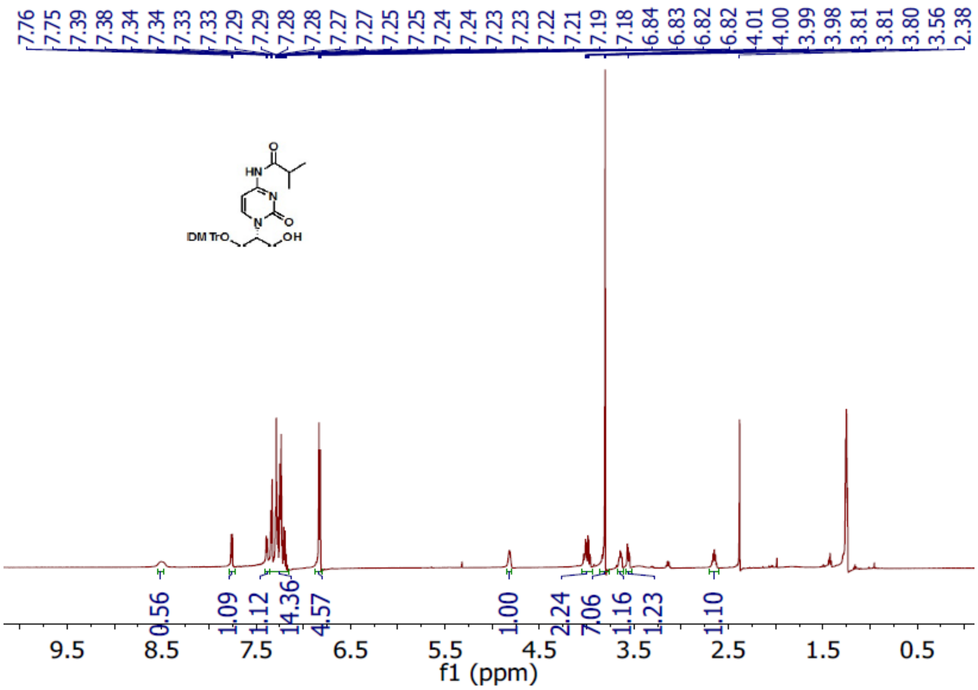

^{1}H NMR spectrum of **21** in $CDCl_3$.

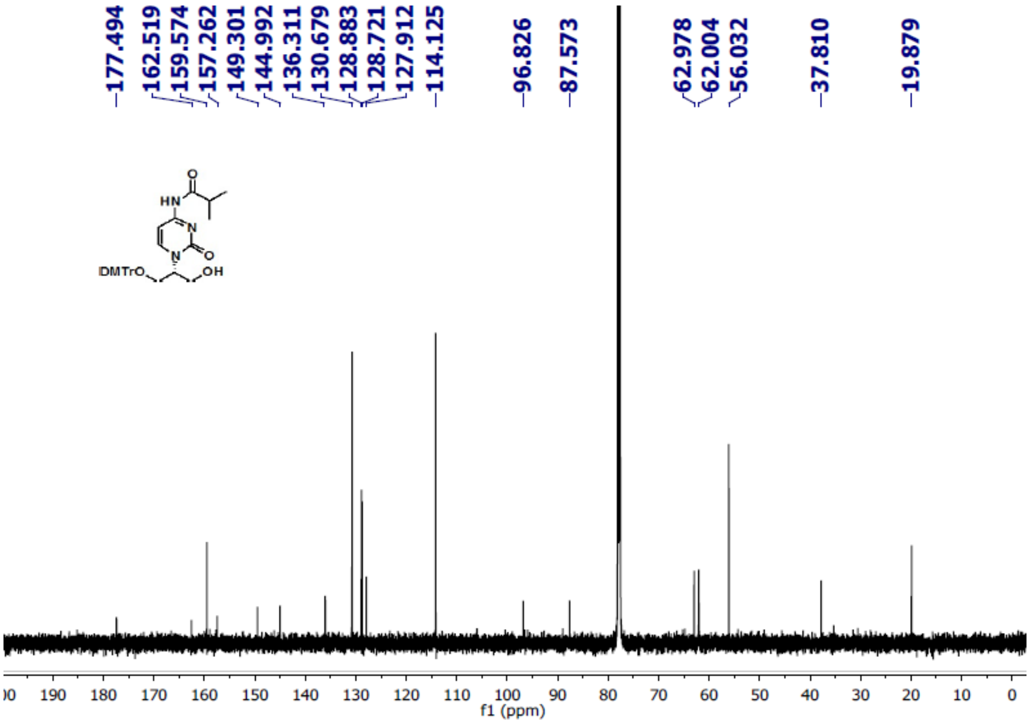

^{13}C NMR spectrum of **21** in $CDCl_3$.

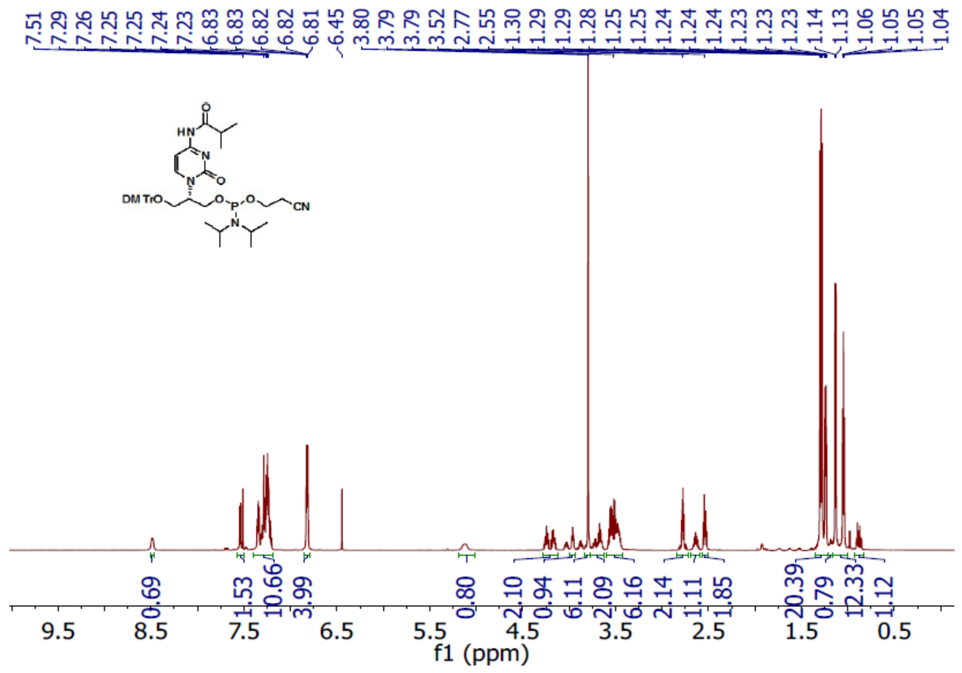

^{1}H NMR spectrum of **22** in $CDCl_3$.

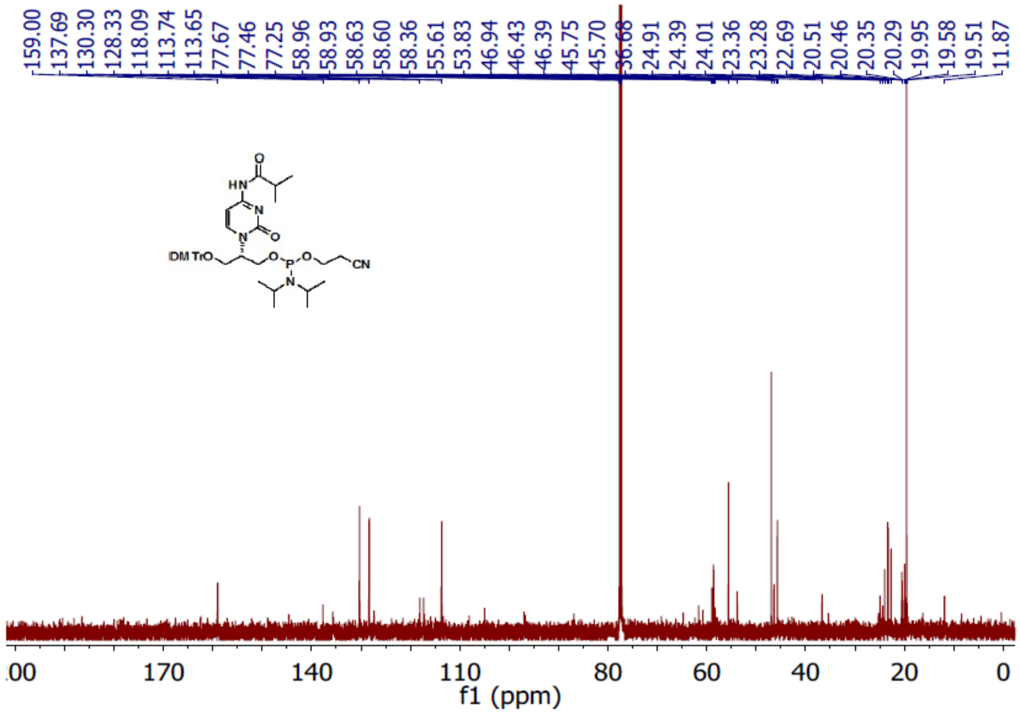

^{13}C NMR spectrum of **22** in $CDCl_3$.

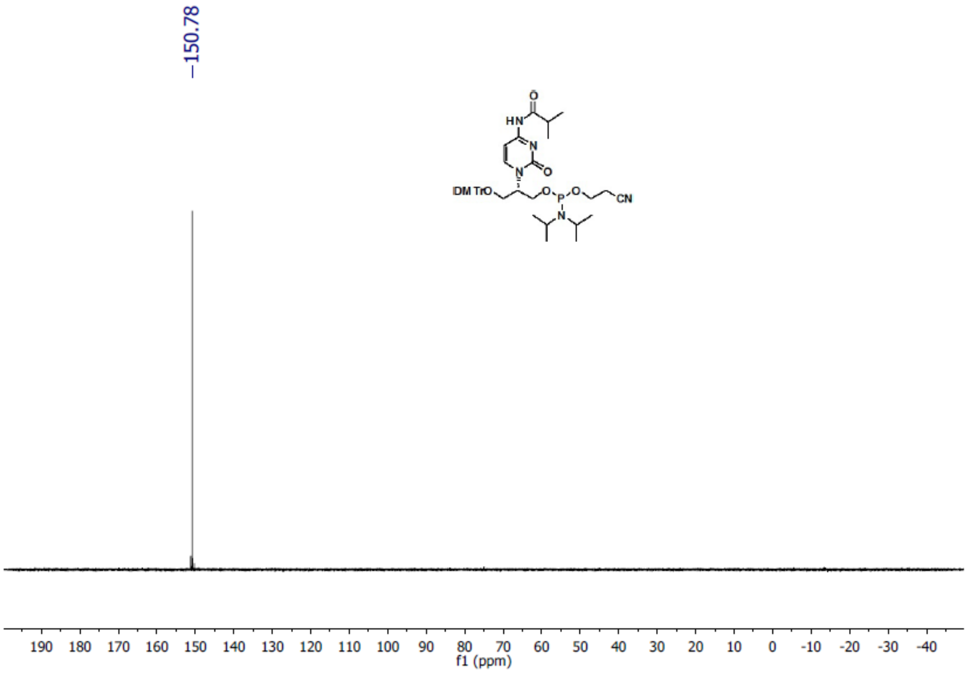

^{31}P NMR spectrum of **22** in $CDCl_3$

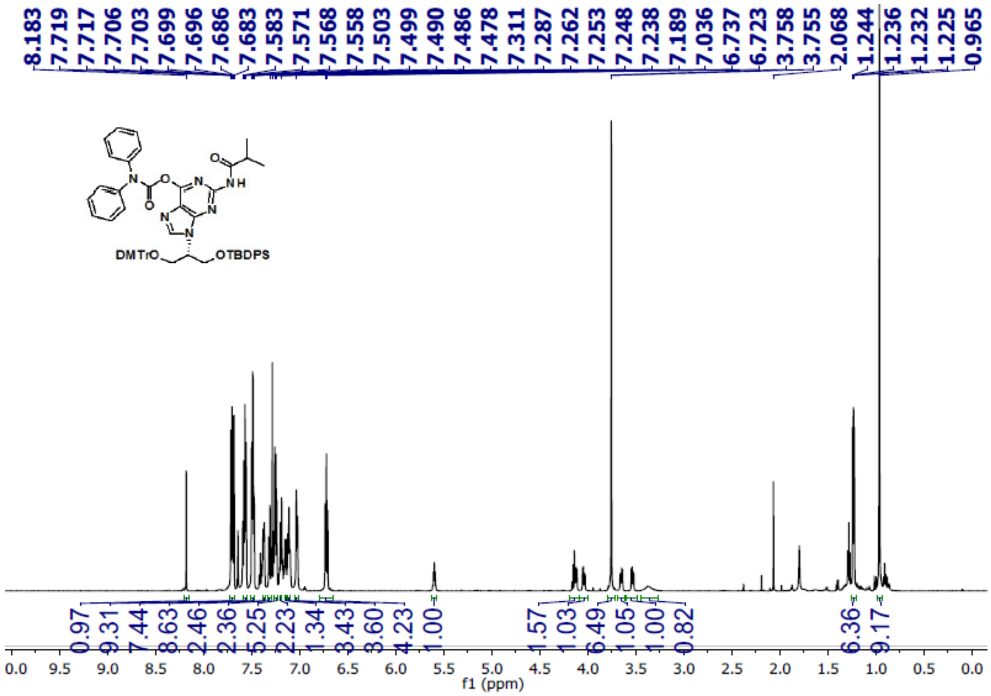

^{1}H NMR spectrum of **23** in $CDCl_3$.

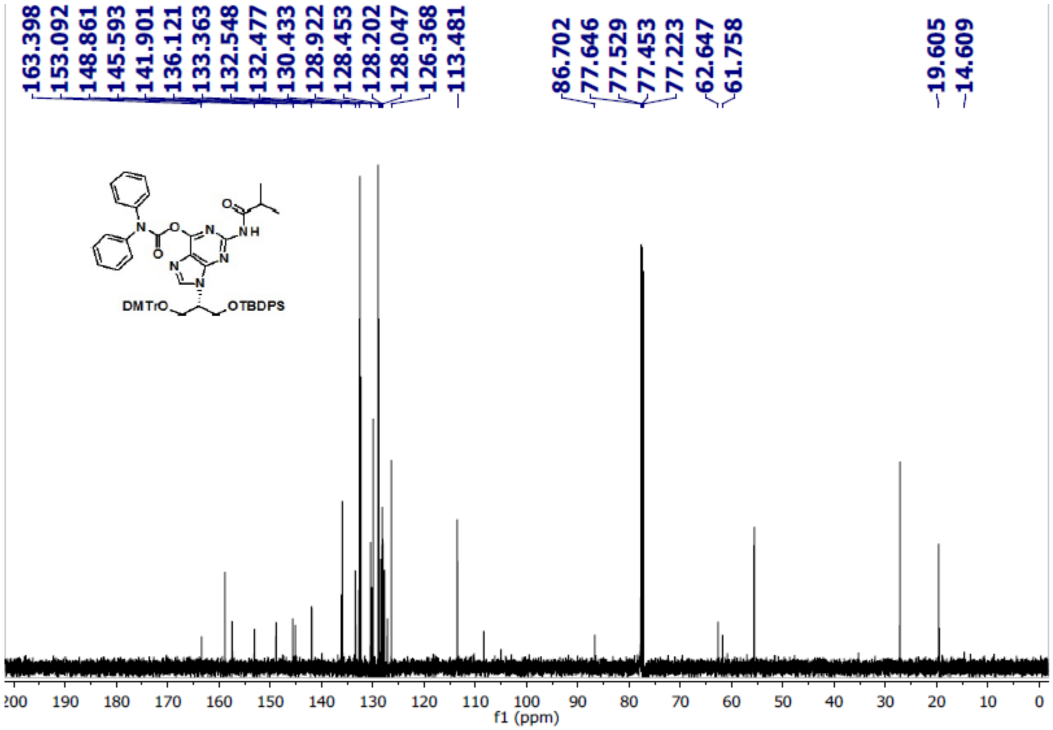

^{13}C NMR spectrum of **23** in $CDCl_3$.

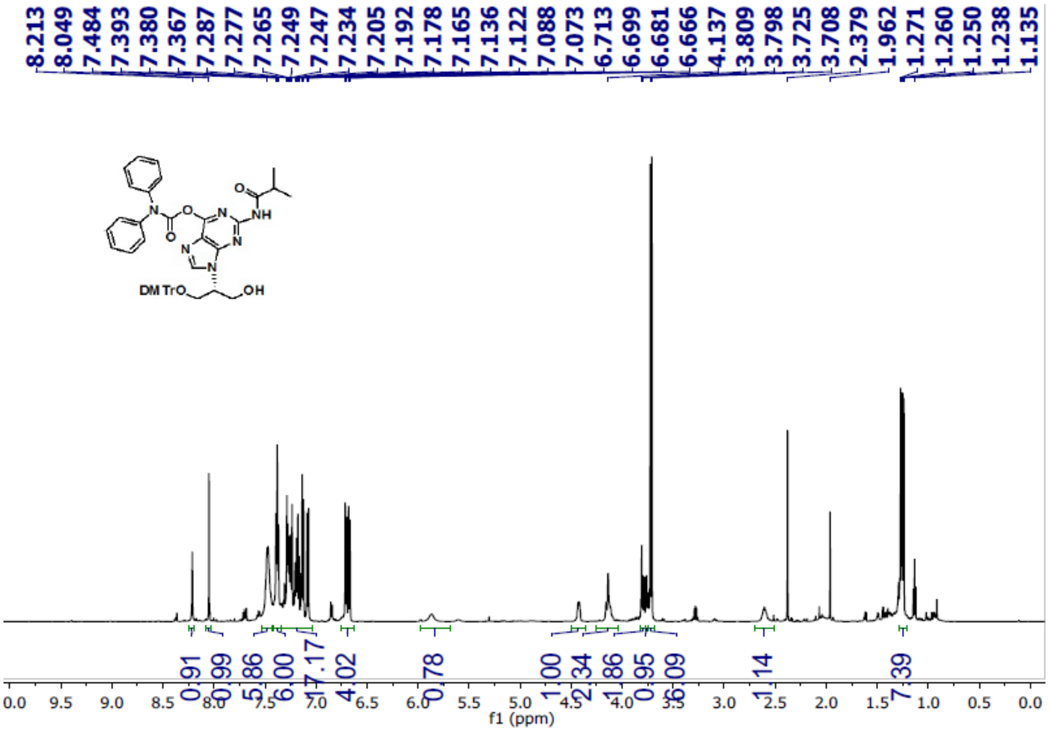

^{1}H NMR spectrum of **24** in $CDCl_3$.

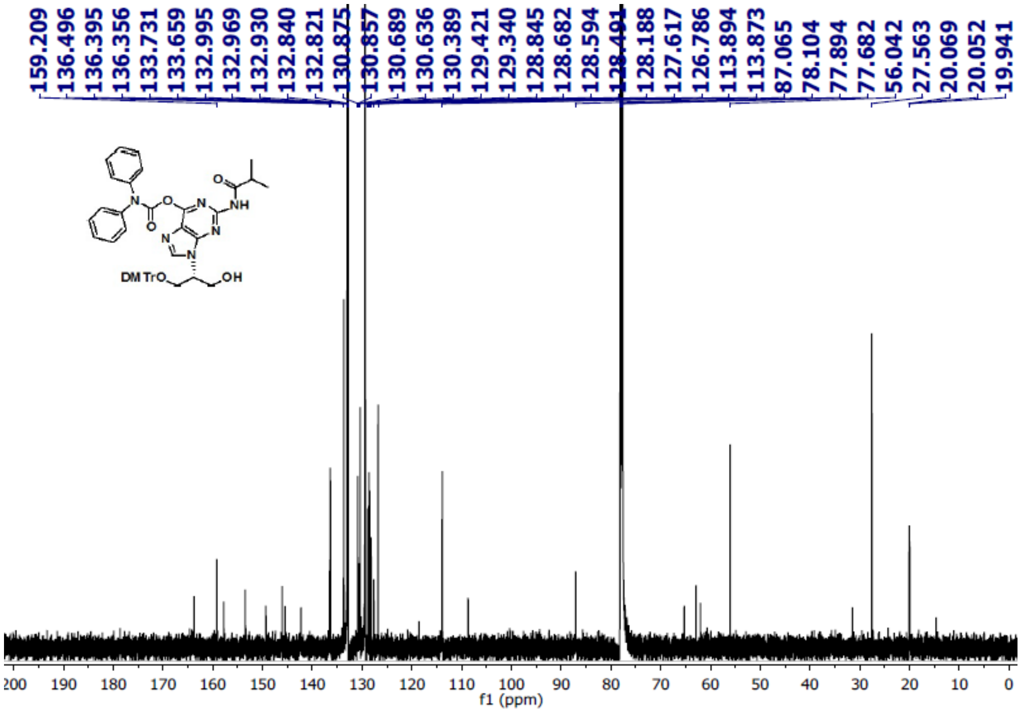

^{13}C NMR spectrum of **24** in $CDCl_3$.

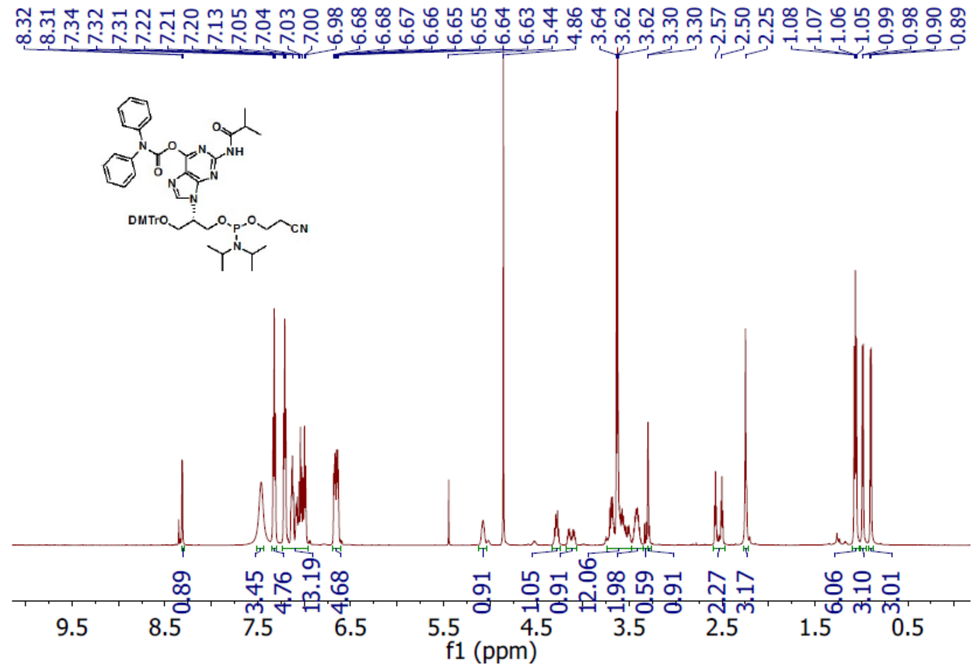

^{1}H NMR spectrum of **25** in CD_3OD.

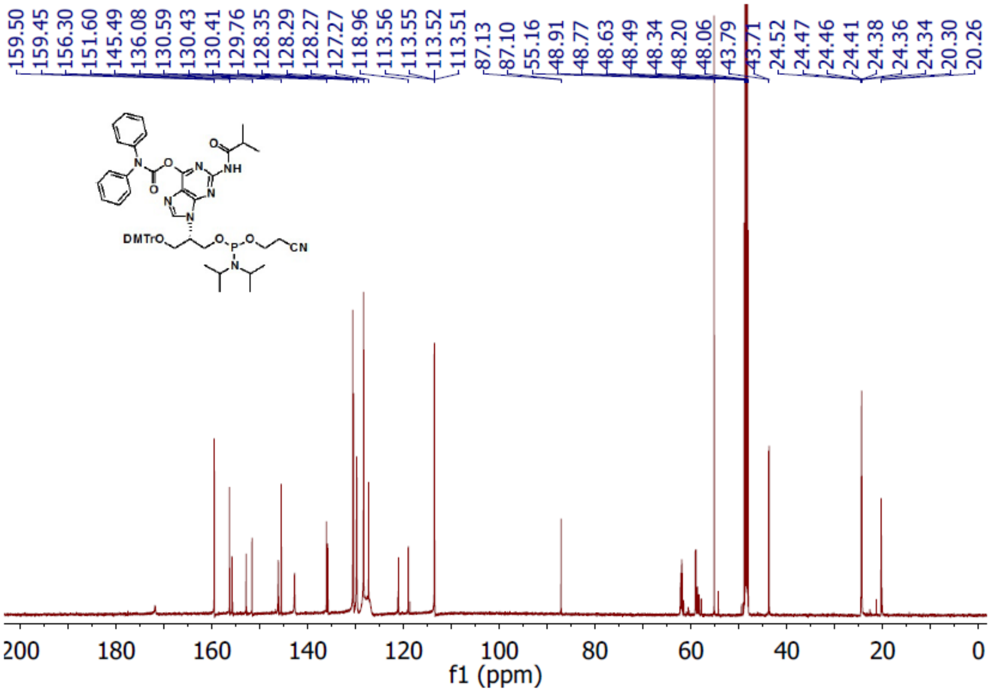

^{13}C NMR spectrum of **25** in CD_3OD.

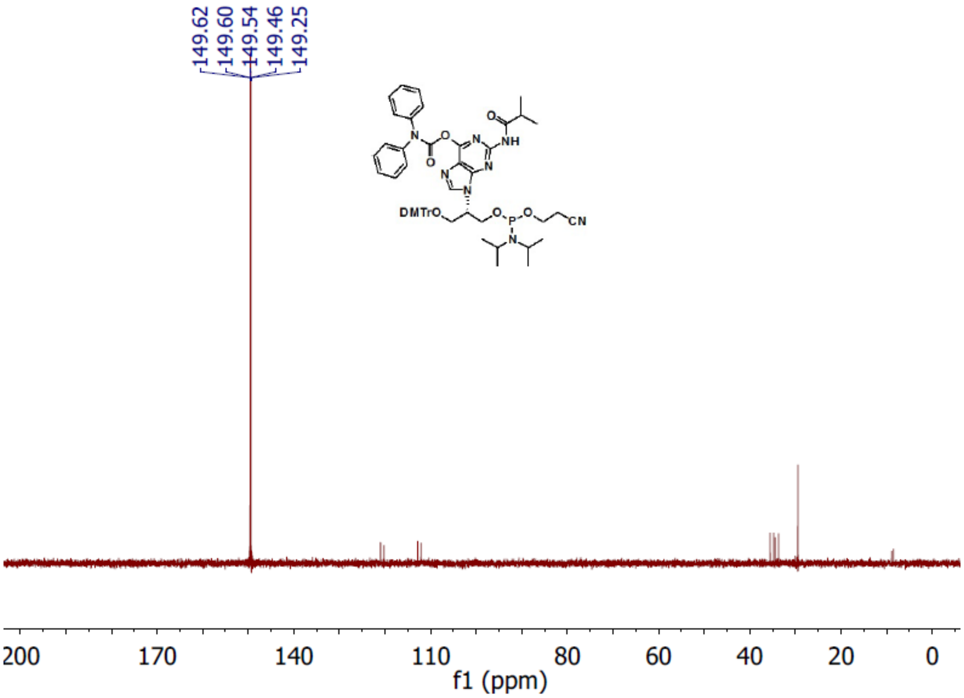

^{31}P NMR spectrum of **25** in CD_3OD.

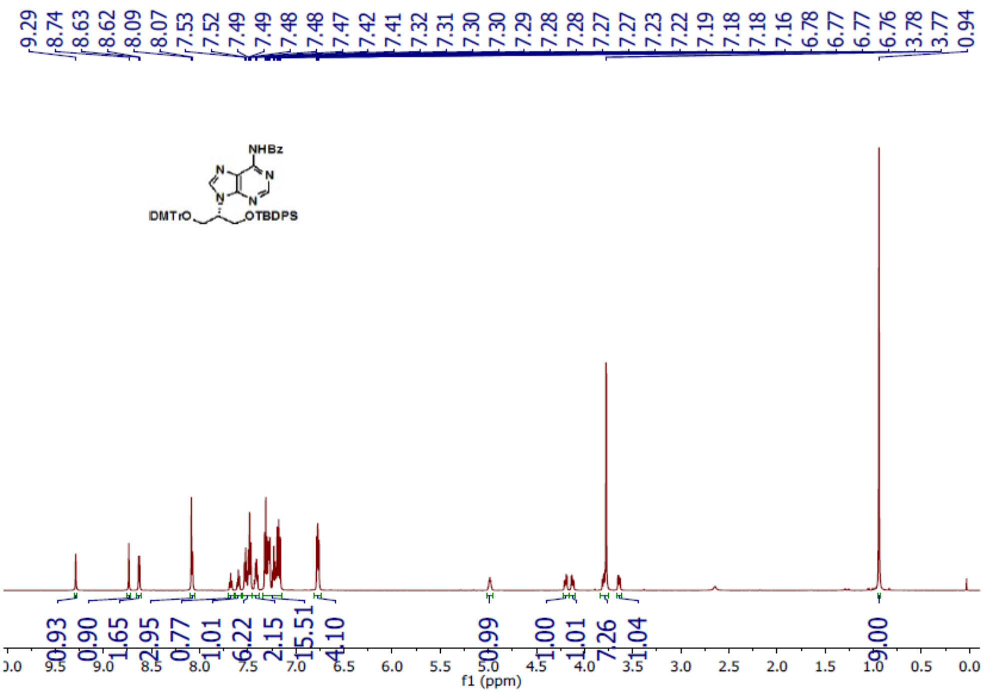

^{1}H NMR spectrum of **26** in $CDCl_3$.

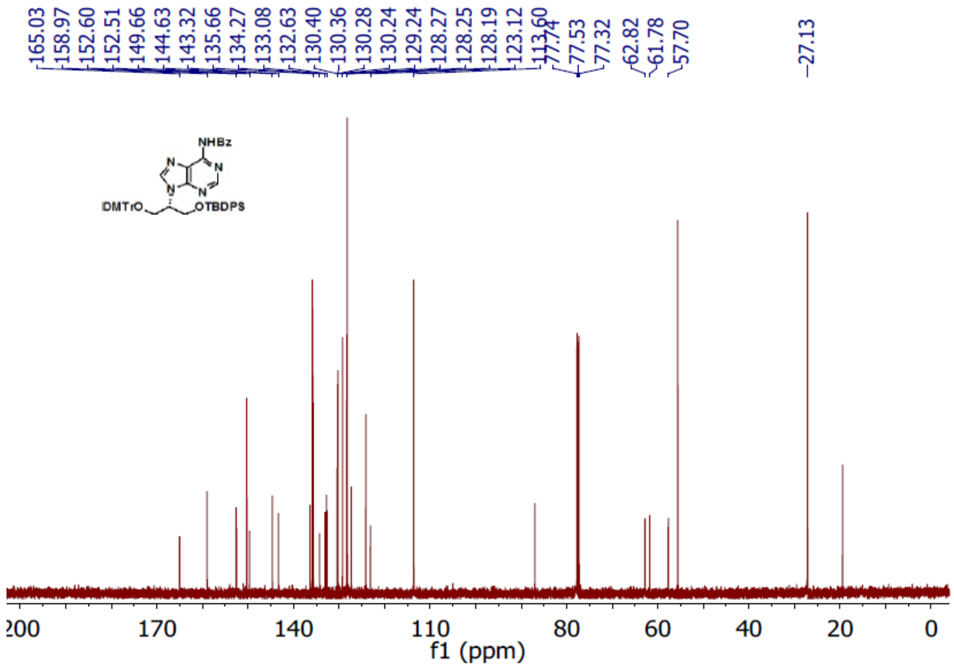

^{13}C NMR spectrum of **26** in $CDCl_3$.

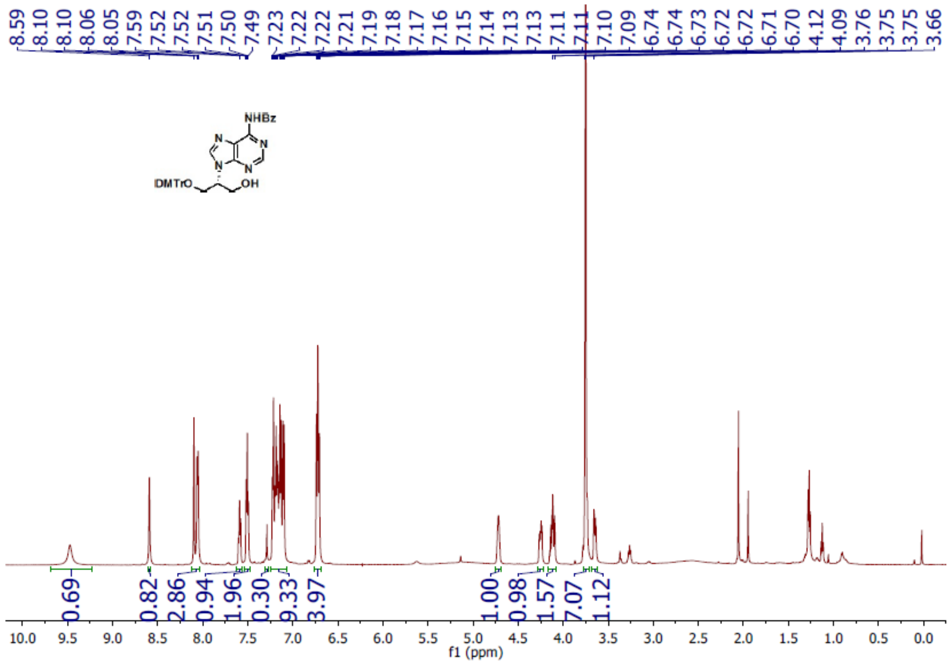

^{1}H NMR spectrum of **27** in $CDCl_3$.

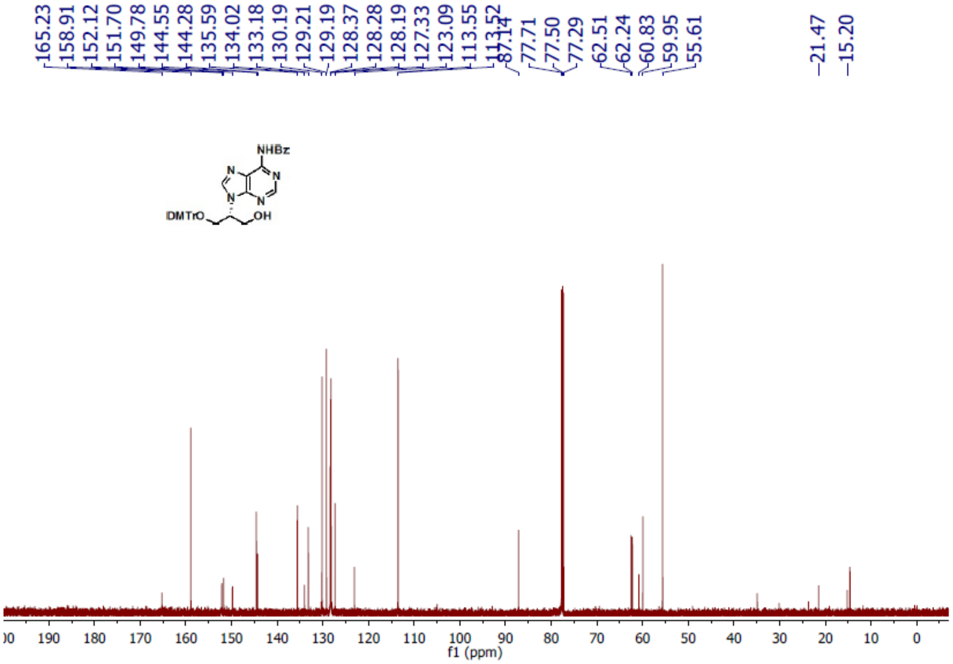

^{13}C NMR spectrum of **27** in $CDCl_3$.

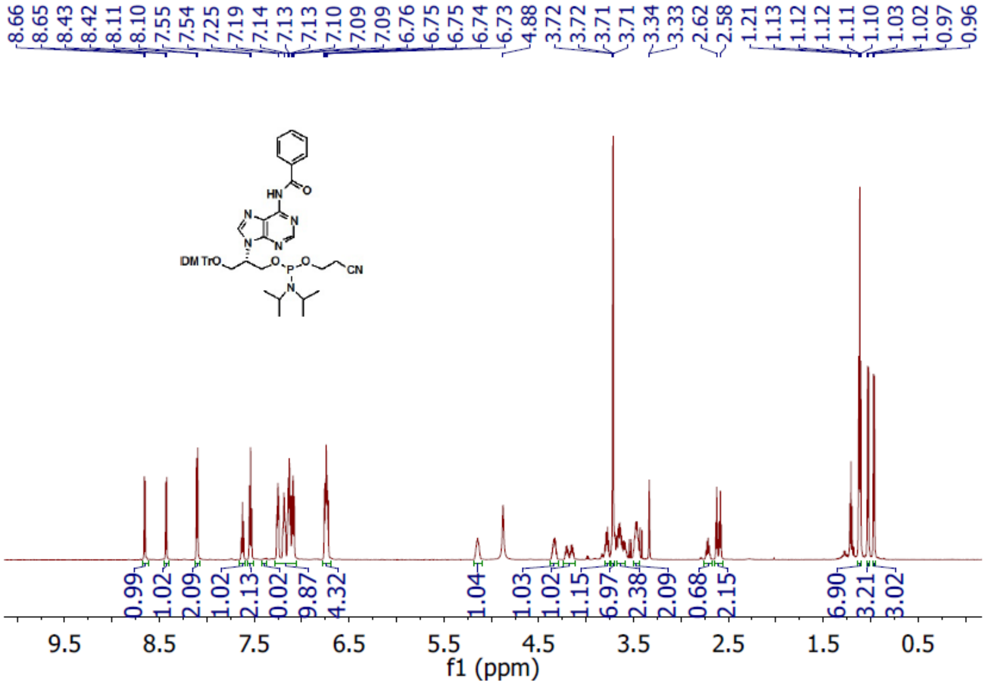

^{1}H NMR spectrum of **28** in CD_3OD.

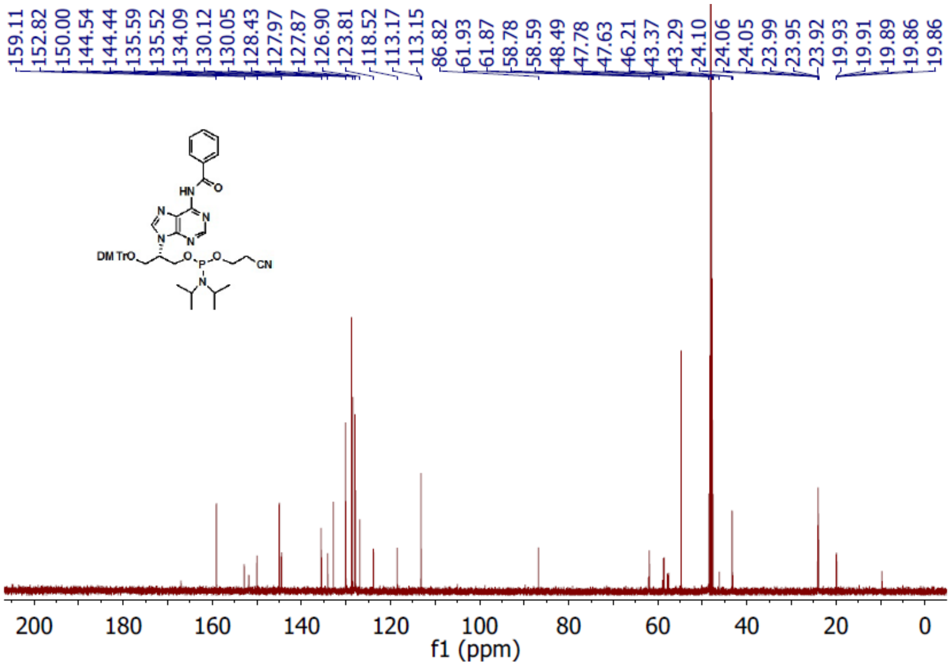

^{13}C NMR spectrum of **28** in CD_3OD.

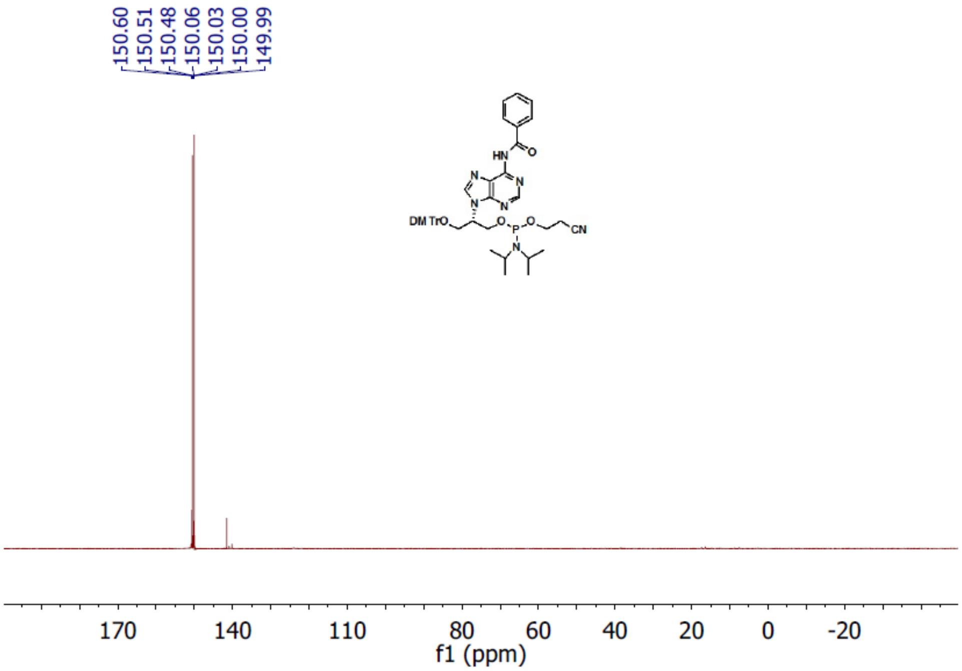

^{31}P NMR spectrum of **28** in CD_3OD.

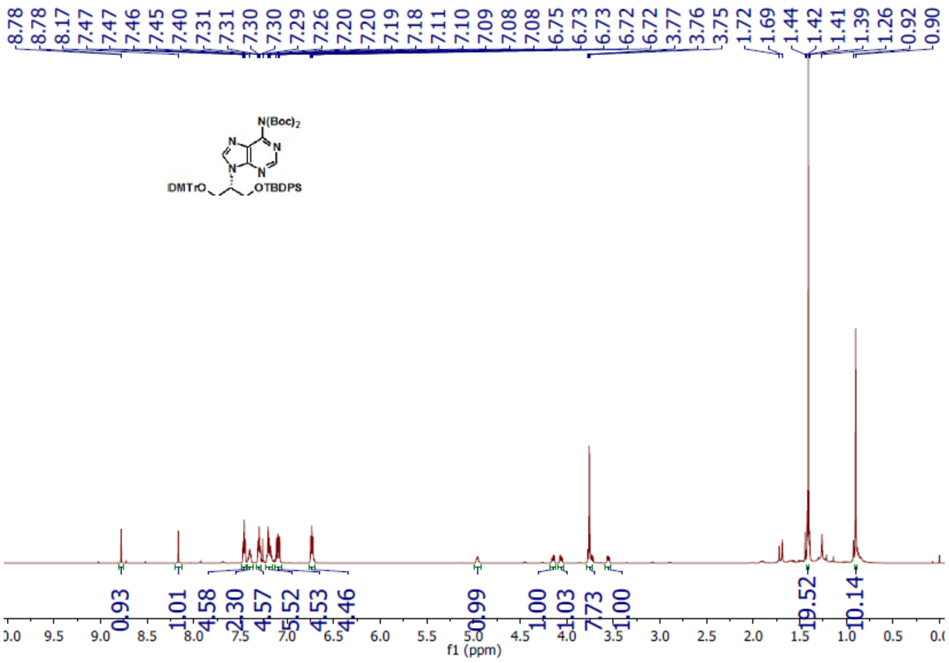

^{1}H NMR spectrum of **29** in $CDCl_3$.

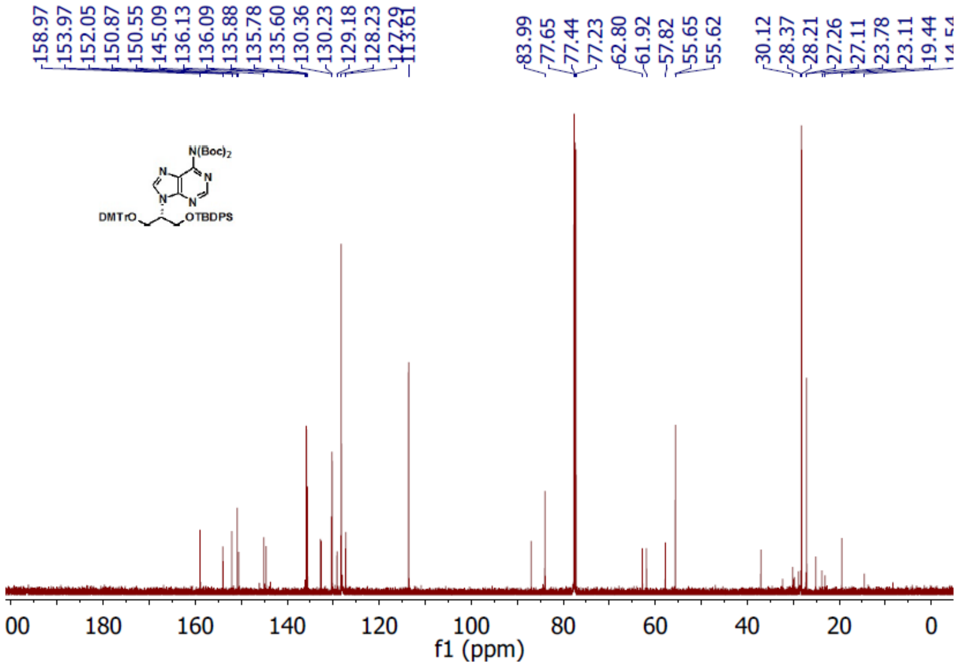

^{13}C NMR spectrum of **29** in $CDCl_3$.

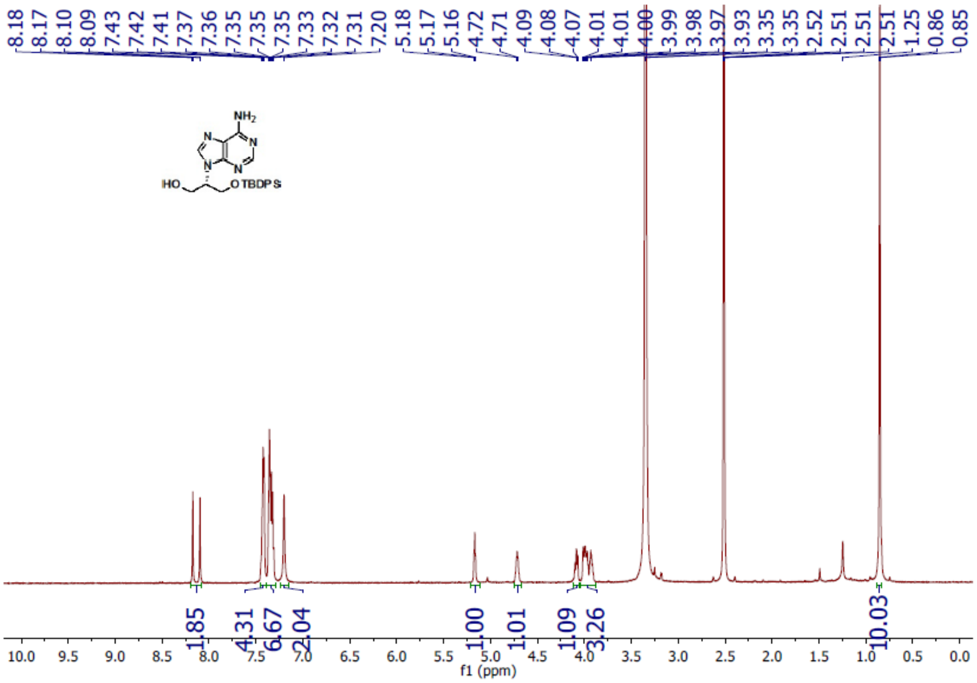

^{1}H NMR spectrum of **33** in DMSO-d_6.

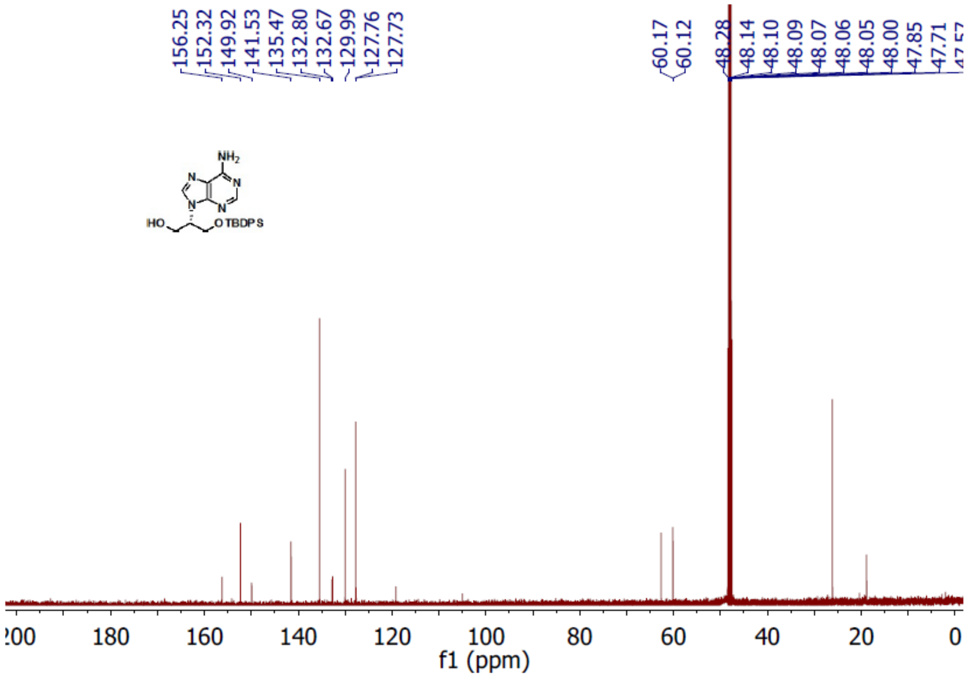

^{13}C NMR spectrum of **33** in DMSO-d_6.

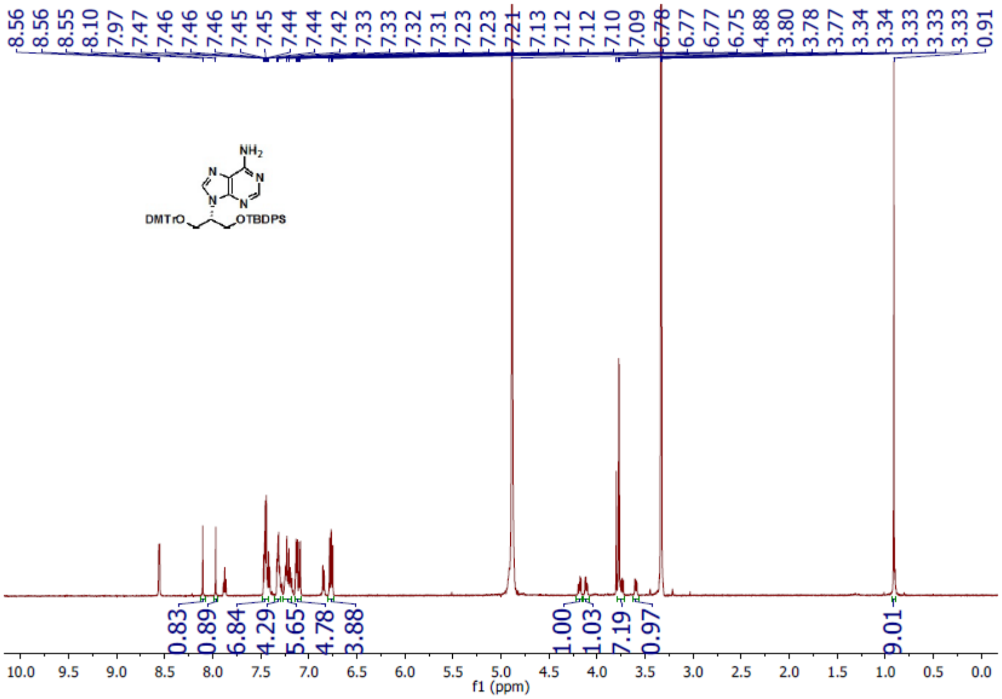

^{1}H NMR spectrum of **34** in CD_3OD.

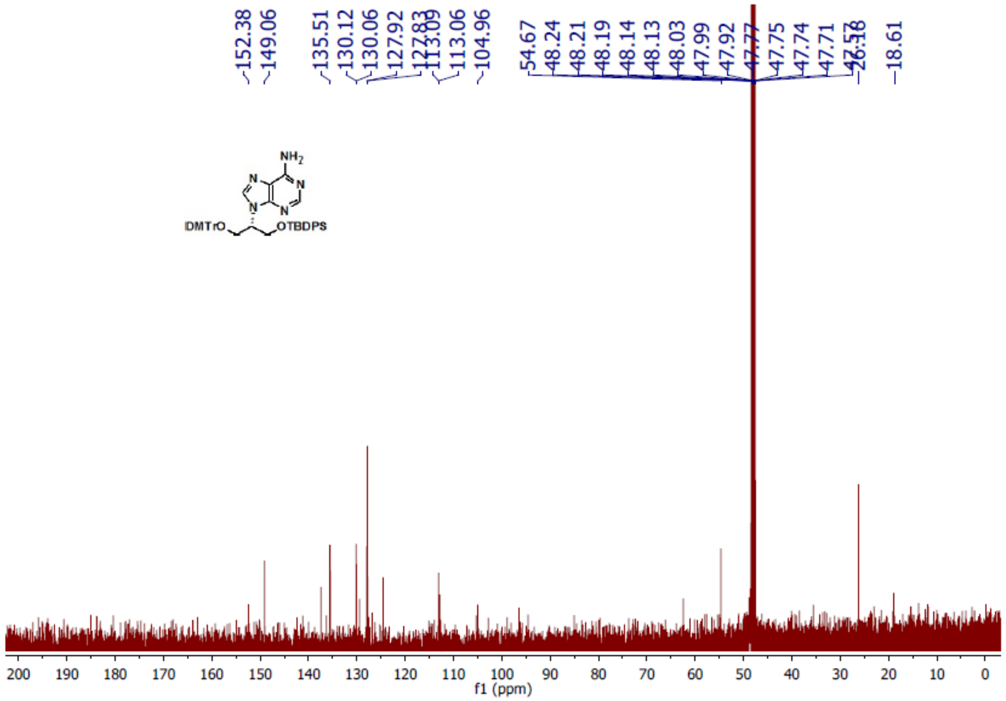

^{13}C NMR spectrum of **34** in CD_3OD.
